# Study on the damage constitutive characteristics of coal-rock composites under uniaxial compression: Influence of prefabricated crack angle and geometric dimensions

**DOI:** 10.1371/journal.pone.0316586

**Published:** 2025-03-05

**Authors:** Qingwen Li, Chuangchuang Pan, Yuqi Zhong, Wenxia Li, Ling Li, Fanfan Nie, Jiabo Chen

**Affiliations:** School of Civil and Architectural Engineering, Liaoning University of Technology, Jinzhou, China; Shenyang Jianzhu University, CHINA

## Abstract

In coal mining environments with complex topographic and geological conditions, the presence of primary cracks in the rock strata of the upper mining airspace is critical to mine safety, especially when roof slabs are broken. Cracked roof slabs not only increase risks during mining but also make mining operations more challenging. Therefore, studying the initial damage state of the roof rock formation is great significance. In this study, the effects of different prefabricated crack sizes and inclination angles on the mechanical properties of the coal-rock composite containing cracks were analyzed through the uniaxial compression experiments and PFC^2D^ numerical simulations. The results show that the peak strength and elastic modulus of the coal-rock composites fall between those of pure coal and sandstone, while the macroscopic mechanical parameters of crack-containing composites are significantly lower than those of non-crack-containing composites. Coal-rock composites with different crack characteristics exhibited different mechanical properties, with their damage modes were caused by the combined effects of tensile and shear damage. The increase in crack inclination altered the crack extension path, and the final damage of the specimen manifested first in the upper part, then the middle part, and ultimately in the lower part of the coal body, with tension-induced bulk damage being the dominant failure mode. Analysis of the radial cumulative map revealed that cracks primarily extended along 90° and 270° directions, indicating a strong tendency for crack propagation under axial pressure. The damage evolution curves indicate a nonlinear relationship between the damage factor and strain. While increased crack inclination enhances the compressive performance of coal-rock composites, it simultaneously accelerates structural destabilization. These findings offer theoretical insights into the damage mechanisms of coal-rock composites with cracks, serving as valuable references for coal mining safety.

## 1 Introduction

In coal mining operations, numerous coal pillars are typically left to stabilize the overlying rock layer in the extraction zone, forming a coal-rock composite structure. When neighboring coal seams have narrow layer spacing, cracks in the surrounding rock above the lower coal seam increase the likelihood of collapses or toppling, significantly influencing the development of impact ground pressure. Thus, studying the mechanical properties of coal-rock composites requires careful consideration of the roof rock layer's initial damage state. This consideration is critical for designing mine support structures and provides a robust theoretical foundation for engineering applications [1–4].

Numerous scholars have examined the mechanical properties and crack evolution of prefabricated cracks in coal-rock masses. Chen et al. [5] observed that penetrating cracks between coal and rock layers significantly influence the mechanical properties of coal-rock systems. The experiments revealed that crack length and angle play a critical role in strength, energy evolution, and destabilization mechanisms. Zhao et al. [6] studied the strength-deformation characteristics and crack extension laws in cracked rock masses under combined water and stress conditions. They found that increasing water pressure transformed crack-bearing rock masses from ductile to brittle damage, with crack angle significantly affecting mechanical properties and damage morphology. Wang et al. [7] used CT scanning and PFC simulations to analyze crack evolution in coal masses containing prefabricated cracks under varying perimeter pressures. They observed that increasing perimeter pressure reduced crack initiation angles and shifted damage modes from tensile-shear to shear failure. Their proposed stress-strength factor formula closely aligned with experimental results. Li et al. [8] examined how prefabricated crack angles affect mechanical properties, damage modes, and acoustic-thermal characteristics of coal specimens through uniaxial compression tests. They found that crack angle significantly influenced peak stress and strain, revealing crack expansion through infrared thermal imaging and acoustic emission analysis. Wang et al. [9] analyzed the mechanical properties of granite with single penetrating cracks. They reported that increasing crack angle significantly reduced compressive strength, damage stress, and elastic modulus, with damage modes transitioning from crack-surface failure to sliding failure along the crack. Li et al. [10] analyzed the effects of various parallel pre-cracking forms on the mechanical properties of coal-rock composites using PFC^2D^ numerical simulations. They found that parallel pre-cracking significantly reduced peak strength, peak strain, elastic modulus, and crack initiation stress, identifying three primary crack initiation modes. Ma et al. [11] examined the mechanical behavior and failure mechanisms of coal-rock composites with different crack angles using uniaxial compression tests and numerical simulations. They found that crack angle significantly influenced strength, deformation, and damage modes, and proposed a failure criterion consistent with experimental results. Chen et al. [12] discovered that water-rock interactions accelerated crack expansion between coal and rock layers, resulting in mechanical property degradation and structural instability. Crack width and soaking time significantly influenced strength, energy characteristics, and damage mechanisms, posing risks to coal mining safety. Zhang et al. [13] employed RFPA^2D^ software to simulate coal-rock composites with coal-sustained joints. They found that joint angle and height ratio significantly influenced uniaxial compressive strength, acoustic emission characteristics, and damage modes. The study proposed that acoustic emission energy accumulation curves could serve as precursors for failure. Li et al. [14] performed dynamic impact tests on pre-cracked coal-rock composites using a split Hopkinson pressure bar. They observed that crack location and angle significantly influenced strength and failure modes, with the most pronounced effects occurring at 30°. They further noted that cracks altered stress concentration and stability. Lv et al. [15] examined the mechanical properties, acoustic emission behavior, and damage modes of coal-rock composites with a single prefabricated non-penetrating crack through theoretical analysis, experiments, and numerical simulations. They concluded that crack angle significantly influenced unconfined compressive strength and failure modes, with strength reaching its lowest at 45°. Wang et al. [16] investigated how layering and prefabricated cracks influence cracking characteristics and breakage mechanisms in black shale using Brazilian splitting tests. They determined that prefabricated cracks weakened specimen strength, while crack initiation and expansion paths were significantly affected by layering angles and crack orientations. The study summarized five failure modes and two acoustic emission counting features. Li et al. [17] investigated how various defect types influence crack initiation, propagation, and damage modes in rock specimens through experiments and numerical simulations. They observed that wing-like cracks originated at defect tips and that crack agglomeration intensified as defect numbers increased. The study summarized the characteristics of cleavage and shear damage in rocks. Zhao et al. [18] explored the cracking and stress-strain behavior of rock-like materials with two defects using uniaxial compression tests and strain analysis. They found that crack merging modes were strongly correlated with defect geometries and that local strain concentration revealed crack development processes. Zeng et al. [19] developed a stable water pressure testing system to study fracture characteristics and acoustic emission behavior of fractured rock under 1 MPa water pressure coupled with stress. Their results indicated that water pressure significantly influenced peak strength, crack extension paths, and acoustic emission characteristics. Wang et al. [20,21] investigated the effect of CO_2_ fracturing on rocks and proposed a fractal damage calculation model. The experiments show that the static load guides the fracture direction, and the dynamic load affects the number and morphology of fracture, which provides a new support for the design of drilling vibration prevention and the study of the damage intrinsic properties of coal rock. Li et al. [22] proposed a two-dimensional eigenstructural model to effectively describe the nonlinear stress-strain behavior of rocks, which provides theoretical support for the damage analysis of coal-rock composites.

In summary, numerous studies on coal-rock composites have primarily focused on the mechanical properties and crack evolution characteristics influenced by prefabricated crack angles and sizes in single materials or interfaces [23,24]. However, the effects of internal cracks within the rock mass have received less attention. This study integrates laboratory experiments with DEM numerical simulations to model the initial damage state of the top rock layer by varying the geometrical parameters of prefabricated cracks within the rock. The results examine the damage modes and evolution processes of cracked coal-rock composites and establish a crack damage constitutive equation, offering theoretical support for stability assessments of coal-rock composites in coal mining.

## 1 Uniaxial compression experiment

### 1.1 Specimen preparation

In this study, pure coal, sandstone, and coal-rock composites were chosen as the experimental materials. The specimens were machined into standard cylindrical shapes with a diameter of 50 mm and a height of 100 mm. The pure coal specimens were collected from the Hepan coal mine located in Shenmu County, Shanxi Province. After on-site sampling, the specimens were cut and polished to prepare standard samples. The sandstone specimens were prepared by first extracting a core from a rock slab using a core drilling machine, followed by processing into a standard cylinder using a stone grinding machine. To maintain uniformity in structure and composition, all sandstone specimens were obtained from the same batch of rock slabs.

The preparation steps for the coal-rock composites are as follows: the treated sandstone and pure coal were cut transversely along the center and divided equally into two specimens of the same size. The cutting surfaces were sandpapered to ensure smoothness and uniformity. Subsequently, high-strength mica glue was used to bond the polished coal-rock interface, ensuring a tight fit, and the specimens were processed into a φ50 mm ×  100 mm composite cylinder. After bonding, the composite specimens were placed in a cool environment for 24 hours to ensure adequate curing. The end faces of all specimens were finely polished to control non-parallelism and non-perpendicularity within 0.02 mm [25].

In addition, prefabricated cracks in the coal-rock composites were generated by wire-cutting. According to Yuan Chao [26] and Zhu et al. [27], the crack angle of approximately 45° has a greater effect on the strength of the specimen, while the effect is less when it is close to 90°. Therefore, the angles of the prefabricated cracks were chosen to be 45° and 90° for this experiment, and the size of the cracks was set to be 25 mm in length and 1.5 mm in width. To minimize the variability in experimental results, each specimen was subjected to three repetitions of the experiment, and the dimensions and mass of the specimens were accurately measured using vernier calipers and electronic scales. The physical parameters obtained are detailed in Table 1.

**Table 1:** Statistics of test results.

Test specimen	Sample label	Diameter/mm	Height/mm	Crack angle/°	Density/(g·cm^-3^)	Peak stress *σ*/MPa		Peak strain *ε*/10^-2^		Elastic modulus *E*/GPa	
						Experimental value	Average value	Experimental value	Average value	Experimental value	Average value
Sandstone	YS-1	50.02	100.10	/	2.41	39.37	39.63	1.20	1.24	4.91	4.82
	YS-2	50.04	100.06		2.43	37.83		1.23		4.76	
	YS-3	50.02	100.00		2.43	41.68		1.29		4.80	
Coal-rock composite	MY-1	49.09	100.04	/	1.91	24.12	23.30	1.32	1.23	2.53	2.64
	MY-2	49.14	100.04		1.94	25.56		1.22		2.81	
	MY-3	50.00	100.16		1.86	20.22		1.15		2.59	
	MYS-45-1	49.1	100.08	45	1.91	16.50	14.59	1.40	1.15	2.23	2.23
		MYS-45-2	49.09	100.08		1.89	12.88		0.99		2.34
		MYS-45-3	49.08	100.00		1.89	14.38		1.05		2.13
	MYS-90-1	49.06	100.00	90	1.86	22.92	23.48	1.29	1.32	2.43	2.51
		MYS-90-2	49.12	100.04		1.87	25.07		1.43		2.55
		MYS-90-3	49.12	100.08		1.88	22.45		1.26		2.54
Coal	CC-1	50.06	100.02	/	1.34	15.61	16.28	1.17	1.34	1.90	1.82
	CC-2	49.14	100.08		1.32	17.57		1.22		1.85	
	CC-3	50.06	100.03		1.32	15.64		1.34		1.70	

### 1.2 Experimental results

The uniaxial compression experiments were conducted using the WDW-300 microcomputer-controlled universal testing machine produced by Changchun Kexin Co., Ltd. The displacement control method was employed for loading, with the loading rate of 0.12 mm/min [13].

**Fig 1:**
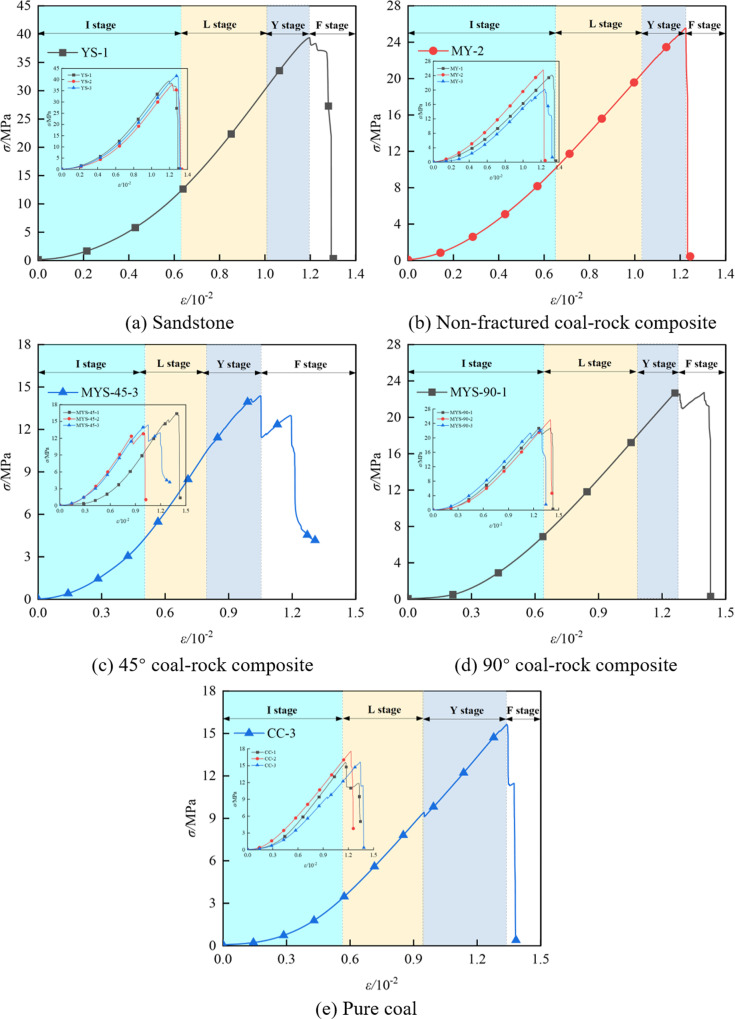
Stress-strain curves of uniaxial experiment.

The stress-strain curves obtained from the experiments are shown in Fig 1. One stress-strain curve from each group of specimens was selected to classify the stages accordingly. The curves can generally be divided into four stages: the initial compaction stage, the linear elastic deformation stage, the yield stage, and the failure stage. The curve behaves differently at each stage: ① Initial compaction stage (I stage): Due to the presence of primary cracks within the specimen, these cracks close under axial compression. This is represented in the graph as the “concave” portion of the curve, where the stress increases rapidly, but the deformation is relatively small. ② Linear elastic deformation stage (L stage): At this stage, the relationship between stress and strain follows Hooke’s law, meaning stress is proportional to strain, and the figure shows a linear relationship. ③ Yield stage (Y stage): When the stress reaches a certain value, the material enters the yield stage. At this stage, the specimen begins to undergo plastic deformation, and the deformation becomes irreversible. The figure shows the yield stage up to the peak stress. ④ Failure stage (F stage): At this point, the stress reaches its maximum value, fracture occurs within the material, the specimen is no longer intact, and the stress begins to decrease. The curve in the figure shows the post-peak stage.

The results indicate that sandstone exhibits the highest peak strength and elastic modulus, with average values of 39.63 MPa and 4.82 GPa, respectively. The peak strength and elastic modulus of pure coal rank second, with an average peak strength of 16.28 MPa and an average elastic modulus of 1.82 GPa. The mechanical parameters of the coal-rock composites are between those of sandstone and pure coal, which is consistent with the conclusion obtained in the literature [28]. The average peak strength of the non-crack coal-rock composites is 23.30 MPa, and the average elastic modulus is 2.64 GPa. The analysis shows that the different material properties of coal and sandstone, as well as the weakening effect of the contact interface between coal and rock, may affect the stress transfer and failure mode, leading to differences in the stress-strain curve. The data from the uniaxial compression tests are summarized in Table 1. The letters CC in the table represent pure coal specimens, YS represents sandstone specimens, and MY represents coal-rock composite specimens without prefabricated cracks, e.g., MYS-45-1 represents coal-rock composite specimen No. 1 with a 45° crack, and so on.

## 2 Establishment of numerical model of fractured coal-rock mass

### 2.1 Numerical simulation

The particle flow code (PFC) is a software tool for modeling and analyzing the motion and interactions of particulate media using the discrete element method. The software effectively simulates the microscopic structural characteristics of coal rock mass, thus accurately reflecting their macroscopic mechanical behavior. In PFC^2D^, a variety of rigid particle contact models are provided. In this study, the parallel bond model (PBM) and the smooth joint model (SJM) are primarily used as microscopic contact models to analyze the mechanical properties of coal-rock mass.

**Fig 2:**
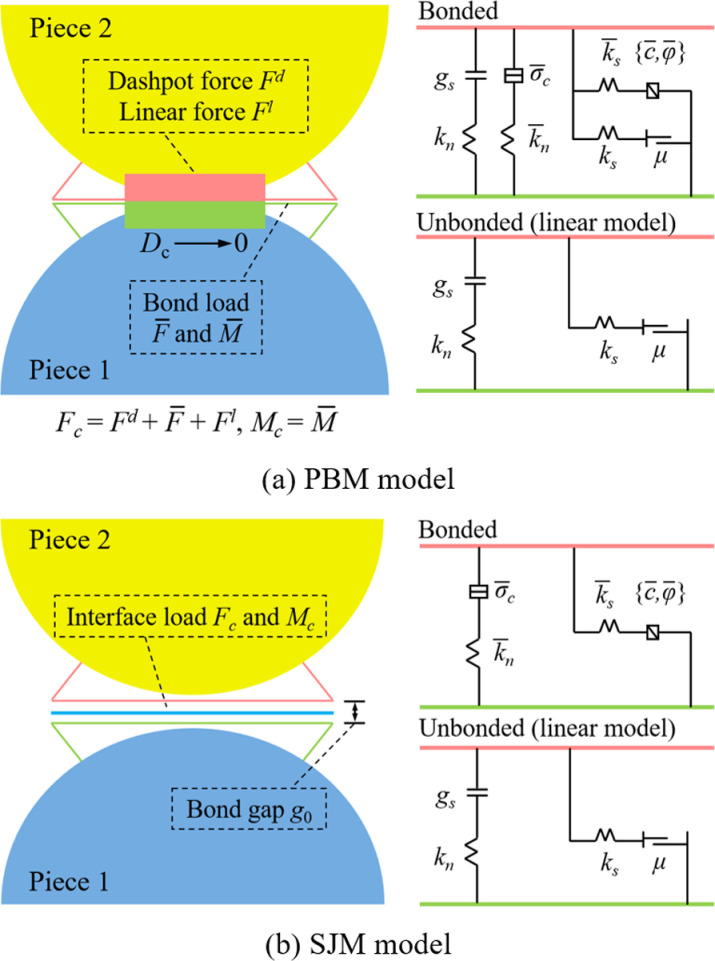
Contact model in PFC.

Fig 2(a) illustrates the PBM model, which is capable of transmitting both force and moment. When the tensile or shear stress exceeds the tensile or shear strength, the particle connections in the PBM model break, thereby more realistically reflecting the mechanical behavior of the coal rock mass. In PFC simulations, the conventional approach to joint simulation is typically achieved by removing the contact on both sides of the joint. However, this method may not adequately model the natural roughness of the crack surface [29]. To address this issue, Deisman [30] proposed the SJM model, as shown in Fig 2(b). The model allows particles to interpenetrate and overlap each other in the direction of the cracks, which effectively improves the simulation accuracy of the mechanical properties of the joints. The model has been widely applied by scholars both domestically and internationally in the simulation study of joint cracks, with results highly consistent with experimental data [31–33].

### 2.2 Calibration of microscopic parameters

**Fig 3:**
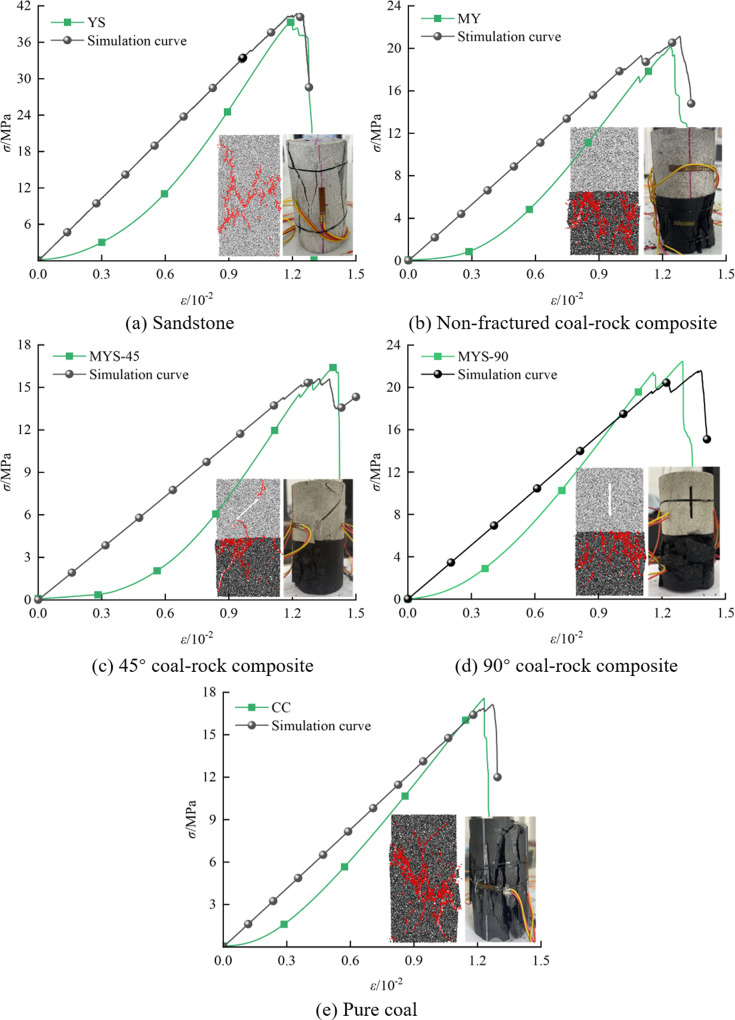
Comparison of numerical simulations and experiments.

PFC simulation uses local contact behavior to describe the macroscopic mechanical properties of materials, allowing numerical experiments to be conducted by setting the geometric and mechanical parameters of particles and bonds [34,35]. Fig 3 shows the comparison of uniaxial compression stress-strain curves obtained from both laboratory experiments and PFC simulations, revealing that the simulation curve closely matches the experimental data. However, in the PFC simulation, due to the rigidity of the particles in the numerical model, the ‘concave’ phenomenon observed in the actual crack compaction process during the initial loading stage is not present. In addition, the peak strength of coal is lower than that of sandstone, indicating that coal fails before sandstone under the same pressure, which is consistent with the results in the literature by Yang et al. [36,37]. During axial compression, the cracks generated inside the coal-rock composites not only expand from the coal body to the sandstone but also cause stress concentration at the crack tip. This stress concentration may cause the crack to further expand, connecting with other primary cracks and eventually leading to shear failure and scattered damage to the coal body. Therefore, the composite structure of sandstone and coal can effectively delay the formation and expansion of coal cracks by increasing the overall structural stability, thereby reducing the risk of composite failure.

Therefore, it is necessary to consider three mesoscopic parameters: pure coal, sandstone, and the joint contact interface. The determination of these three parameters is typically calibrated using the ‘trial and error’ method. After repeated calibration, the simulation data become consistent with the experimental data, and the obtained microscopic parameters are shown in Table 2. The introduction of prefabricated cracks may lead to internal instability, which can eventually cause discrepancies in the stress-strain curves. Therefore, the simulation is based on the average value of the experimental data.

**Table 2:** Numerical modeling fine-scale parameters.

Mesoscopic parameters	Parallel bond model (PBM)	
	Sandstone	Coal
Particle density/(kg/m^3^)	2430	1330
Solid friction factor	0.2	0.3
Particle normal stiffness (N/m)	2.8 × 10^8^	2.8 × 10^8^
Particle tangential stiffness (N/m)	2.8 × 10^8^	2.8 × 10^8^
Effective modulus/GPa	4.0	1.7
Stiffness ratio	2.7	2.9
Parallel bond tensile strength/MPa	36	15
Parallel bond strength/MPa	48	130
Angle of friction/°	60	45
Particle size/mm	0.4 ~ 0.6	
	**Smooth joint model (SJM)**	
Dip angle/°	0	
Size	60	
Normal stiffness/(N/m)	3.49 × 10^7^	
Tangential stiffness/(N/m)	6.5 × 10^7^	
Coefficient of friction	0.3	
Cohesion/GPa	20	
Joint friction angle/°	0.4	

In Table 3, the differences in macroscopic mechanical parameters between the average values obtained from the numerical model and the experimental tests are compared in detail. The results show that the simulated uniaxial compressive strength and elastic modulus closely match the experimental results. The relative errors between the experimental and simulated values were less than 10%. In addition, the failure modes of the two fracture numerical models are essentially the same as the actual failure modes observed in the experimental tests, indicating that the mesoscopic parameters and modeling methods used are suitable for related research.

**Table 3:** Comparison of macro mechanical parameters.

**Test specimen**	**Peak strength *σ*/MPa**			**Peak strain *ε*/10** ^**-2**^		
	**Experimental value**	**Simulation value**	Relative error/%	**Experimental value**	**Simulation value**	**Relative erro/%**
Sandstone	39.63	40.31	1.72	1.24	1.19	4.03
Non-fractured coal-rock composites	23.30	21.13	9.31	1.23	1.28	4.07
45° coal-rock composites	14.59	15.55	6.58	1.15	1.25	8.69
90° coal-rock composites	23.48	21.58	8.09	1.32	1.38	4.55
Pure coal	16.28	17.13	5.22	1.34	1.29	3.73

### 2.3 Simulation scheme

To study the effect of initial roof damage on the impact hazard of coal mining, this paper simplifies and quantifies the roof and coal seam, combining them into a coal-rock composite with a ratio of 1:1. Based on the actual working conditions of the upper rock roof and the lower coal seam at the site, a numerical model of the coal-rock composites with dimensions of φ50 mm ×  100 mm was established using the numerical simulation software PFC^2D^.

**Fig 4:**
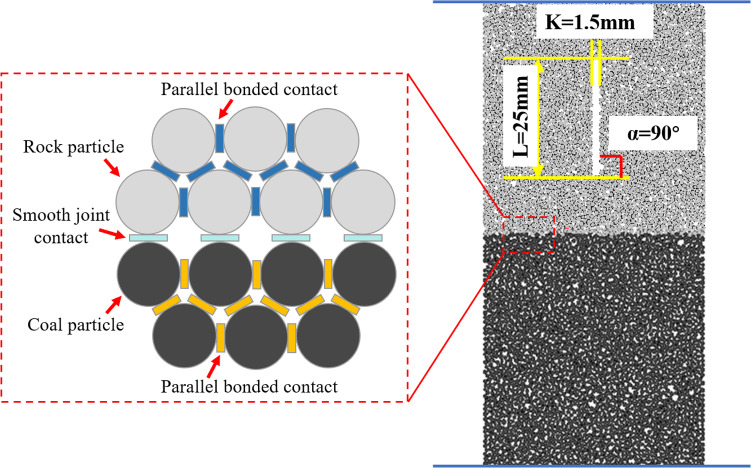
Modelling of 90° coal-rock composite.

In order to ensure that the crack features in the model are consistent with the crack characteristics observed in the laboratory experiments, the prefabricated cracks were generated by removing the particles. The crack length was fixed at *L* =  25 mm, and the crack widths were set at *K* =  1 mm, 1.5 mm, and 2.0 mm. Additionally, the crack angles were selected as 0°, 30°, 45°, 60°, and 90°, based on their common usage in the literature. These angles have been widely adopted in similar studies, such as those by Liu et al., Luo et al., Chen et al., Zhang et al., and Wen et al. [5,38–41], to investigate the mechanical behavior of fractured rock masses under various conditions. As an example, the coal-rock composite with 90° cracks is modeled, and the relevant model is shown in Fig 4.

Uniaxial compression numerical simulation experiment was conducted on the coal-rock composite using a displacement loading control method, with a loading rate set at 0.12 mm/min. The aim was to explore the damage mechanism of the coal-rock composite in the presence of cracks and to analyze the effects of the crack inclination angle and crack width on the composite. The detailed simulation scheme is shown in Table 4. The number *L*25-*K*1-*α*0° indicates that the crack length of the sample is 25 mm, the crack width is 1 mm, and the inclination angle is 0°.

**Table 4:** Mesoscopic simulation scheme.

Crack length *L*/mm	Crack width *K*/mm	Prefabricated crack angle *α*/°	Specimen number
25	1	0	*L*25-*K*1-*α*0°
		30	*L*25-*K*1-*α*30°
		45	*L*25-*K*1-*α*45°
		60	*L*25-*K*1-*α*60°
		90	*L*25-*K*1-*α*90°
	1.5	0	*L*25-*K*1.5-*α*0°
		30	*L*25-*K*1.5-*α*30°
		45	*L*25-*K*1.5-*α*45°
		60	*L*25-*K*1.5-*α*60°
		90	*L*25-*K*1.5-*α*90°
	2	0	*L*25-*K*2-*α*0°
		30	*L*25-*K*2-*α*30°
		45	*L*25-*K*2-*α*45°
		60	*L*25-*K*2-*α*60°
		90	*L*25-*K*2-*α*90°

## 3 Macro-micro mechanical characteristics of fractured coal-rock mass

### 3.1 Macroscopic mechanical properties

**Fig 5:**
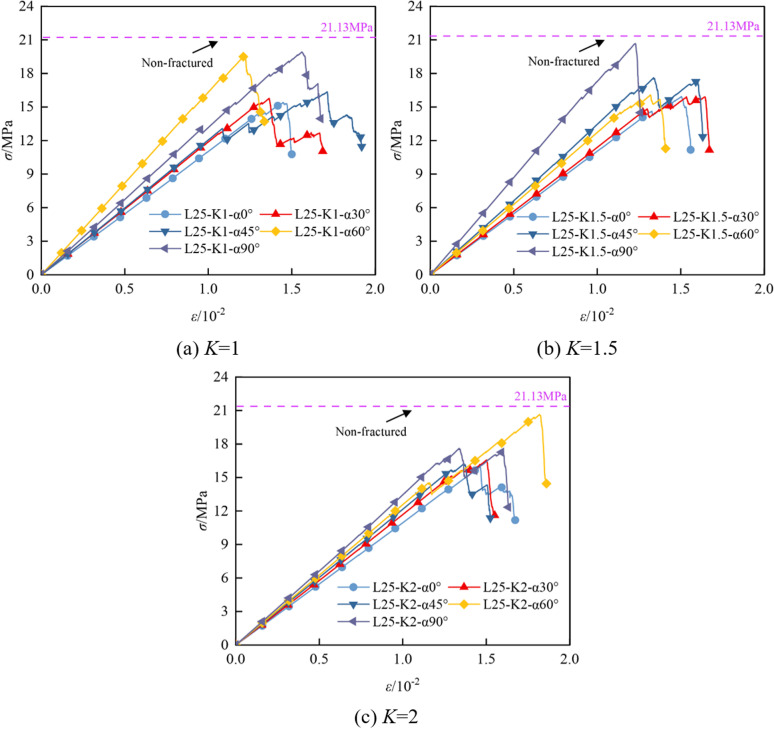
Comparison of characteristic stress-strain curves.

The stress-strain curves under uniaxial compression for the specimens of coal-rock mass with different crack types are shown in Fig 5(a), (b) and (c). Specimens with crack angles of 0°, 30°, 45°, 60°, and 90° exhibited slight oscillations near the peak strength due to crack propagation, and produced jagged horizontal fluctuations in the vicinity of the peak, along with some fluctuations both before and after the peak. This behavior is primarily attributed to local micro-cracks and closure caused by stress concentration at the crack tip, as well as dynamic changes in the microstructure and the redistribution of stress as the specimen approaches failure. These microscopic mechanisms have a certain influence on the macroscopic mechanical properties of the coal and rock mass.

**Fig 6:**
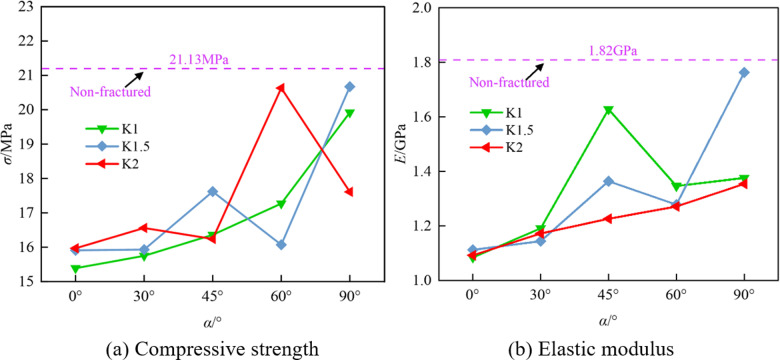
Comparison of different macro-parameters.

Fig 6(a) and (b) show the comparison of macroscopic mechanical parameters of coal and rock specimens with different crack widths under uniaxial compression. It is observed that the macroscopic parameters of the cracked coal-rock composites are lower than those of the non-fractured composites. This is because the fracture, as a defect within the material, reduces the overall continuity and uniformity of the coal-rock mass, leading to a decrease in its mechanical properties. As the fracture angle increases, coal and rock mass specimens with different fracture widths exhibit different mechanical behaviors.

It can be observed that the compressive strength and modulus of elasticity of the cracked coal-rock composites exhibit significant fluctuating trends as the crack width and angle change. Firstly, the compressive strength shows a decreasing trend followed by an increasing trend with the crack angle. For the specimen with *K* =  1 mm, the compressive strength gradually decreases from 15.39 MPa at *α* =  0° to 15.17 MPa at *α* =  30°, then reaches a peak of 16.36 MPa at *α* =  45°, and finally increases to 17.10 MPa at *α* =  90°. The fluctuation in compressive strength intensifies with increasing crack width. For instance, the maximum value of 20.98 MPa is reached at *α* =  60° for *K* =  2 mm. Overall, larger crack widths and angles significantly influence the fluctuation of compressive strength, particularly with an increase in strength at larger angles, which may be related to the change in crack propagation pathways. The modulus of elasticity exhibits a ‘W’-shaped fluctuation trend, which becomes more pronounced, especially as the crack width increases. For the specimen with *K* =  1 mm, the modulus of elasticity decreases from 1.084 GPa at *α* =  0° to 1.148 GPa at *α* =  30°, peaks at 1.628 GPa at *α* =  45°, decreases to 1.346 GPa at *α* =  60°, and finally rises to 1.376 GPa at *α* =  90°. The fluctuation in the modulus of elasticity is more pronounced with *K* =  2 mm, reflecting the complex effect of prefabricated cracks on the mechanical properties at different angles.

Overall, the crack angle and width have a significant effect on the compressive strength and modulus of elasticity of the cracked coal-rock composites, and the presence of cracks alters the internal stress distribution and crack propagation paths, leading to periodic fluctuations in mechanical properties as the crack characteristics change. This provides an important theoretical foundation and guidance for geotechnical engineering design and rock crack prediction. The numerical simulation results are summarized in Table 5.

**Table 5:** Numerical simulation results statistics.

Specimen number	Crack length *L*/mm	Crack width *K*/mm	Prefabricated crack angle α/°	Peak strength *σ*/MPa	Peak strain *ε*/%	Elastic modulus *E*/GPa
*L*25-*K*1-*α*0°	25	1	0	15.39	1.45	1.084
*L*25-*K*1-*α*30°			30	15.75	1.36	1.191
*L*25-*K*1-*α*45°			45	16.36	1.71	1.628
*L*25-*K*1-*α*60°			60	17.27	1.35	1.346
*L*25-*K*1-*α*90°			90	19.92	1.56	1.376
*L*25-*K*1.5-*α*0°		1.5	0	15.91	1.50	1.112
*L*25-*K*1.5-*α*30°			30	15.93	1.53	1.144
*L*25-*K*1.5-*α*45°			45	17.62	1.33	1.364
*L*25-*K*1.5-*α*60°			60	16.07	1.31	1.278
*L*25-*K*1.5-*α*90°			90	20.67	1.22	1.763
*L*25-*K*2-*α*0°		2	0	15.97	1.46	1.092
*L*25-*K*2-*α*30°			30	16.56	1.60	1.172
*L*25-*K*2-*α*45°			45	16.24	1.37	1.226
*L*25-*K*2-*α*60°			60	20.63	1.82	1.271
*L*25-*K*2-*α*90°			90	17.61	1.33	1.354

### 3.2 Micromechanical response

In order to further explore the failure characteristics of coal-rock composites with prefabricated cracks, the meso-cracks are studied in greater detail. According to the failure mode, the meso-cracks can be classified into: ① wing-shaped crack (W); ② coplanar secondary cracks (C); ③ inclined secondary cracks (O) [42–44]. Based on the mode of force failure, the meso-cracks can be divided into: ① tensile crack (T); ② shear crack (S).

**Fig 7:**
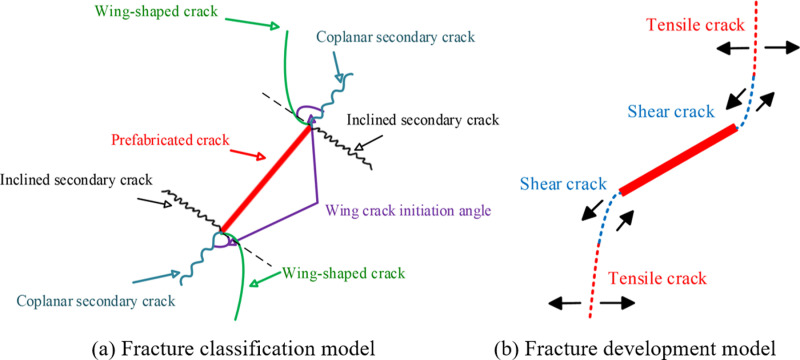
Damage sketches for fracture of precast elements.

**Fig 8:**
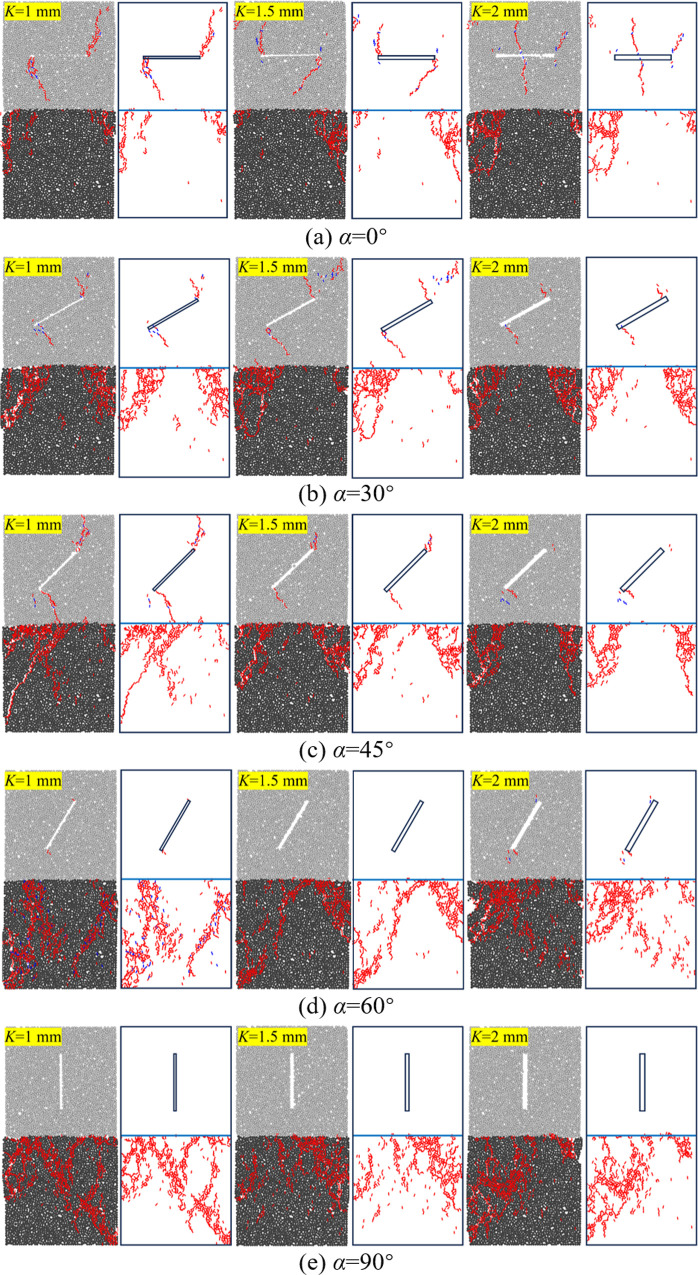
Damage patterns and fracture sketchers.

The failure diagram of the preset crack is shown in Fig 7. The failure mode, overall displacement vector diagram, and crack sketch of different specimens are presented in Fig 8. When the tensile stress and shear stress on the particles exceed the maximum tensile stress and maximum shear stress, respectively, the parallel bond is destroyed, resulting in tensile and shear cracks in the material. This is the crack generation mechanism in the PFC numerical simulation tests. In Fig 8, blue represents shear failure cracks, and red represents tensile failure cracks. Tensile-shear composite cracking occurs both within the rock material and at the contact interface. Tensile cracking predominates within the pure coal material, but as axial compression increases, crack evolution becomes more complex within the specimen. During the simulation, due to differences in the cracking mechanism and the shape of the prefabricated cracks, the cracks within the specimen are generally categorized into three types: S crack, T crack, and S-T mixed crack. By comparing the displacement vector diagram with the crack sketch, it is easier to observe that the tensile crack propagates along the stress direction in a relatively uniform manner in most cases, while secondary cracks form near the main crack zone.

As shown in Fig 8, it can be observed that the damage to coal-rock composites is primarily concentrated in the coal body and the interface, which aligns with the findings in the literature [45]. When the width of the prefabricated crack is kept constant, the upper rock material is damaged along the crack tip when the crack angle is 0° and 30°. With the transfer of stress, the lower coal body also experiences minor tensile failure. As the crack angle increases to 45°, the cracks formed at this point create a through-face as the prefabricated crack tip propagates through the coal-rock interface into the coal body. As the crack angle increases to 60° and 90°, the number of cracks in the upper rock material decreases, and the degree of damage gradually diminishes. However, the lower coal material experiences the opposite effect. The analysis suggests that increasing the crack angle alters the path of crack downward expansion, affecting the lower coal material and potentially inducing new crack formation or increasing the activity of primary cracks in the coal body during the extension process.

Combined with the analysis of the stress-strain curve in Fig 5, it can be seen that under the action of external load, not only tensile cracks are formed within the composite, but shear cracks also develop. This indicates that the instability and failure of the sample are caused by the combined action of tensile and shear forces, rather than a single failure mechanism. Therefore, when evaluating the structural stability of the sample, the interaction between shear and tensile failure must be considered. Focusing solely on one failure mechanism may result in an overestimation of the structural safety factor, thereby posing a greater safety risk.

## 4 Analysis of crack initiation stress and damage evolution of fractured coal-rock mass

### 4.1. Analysis of crack initiation stress of fractured coal-rock mass

**Fig 9:**
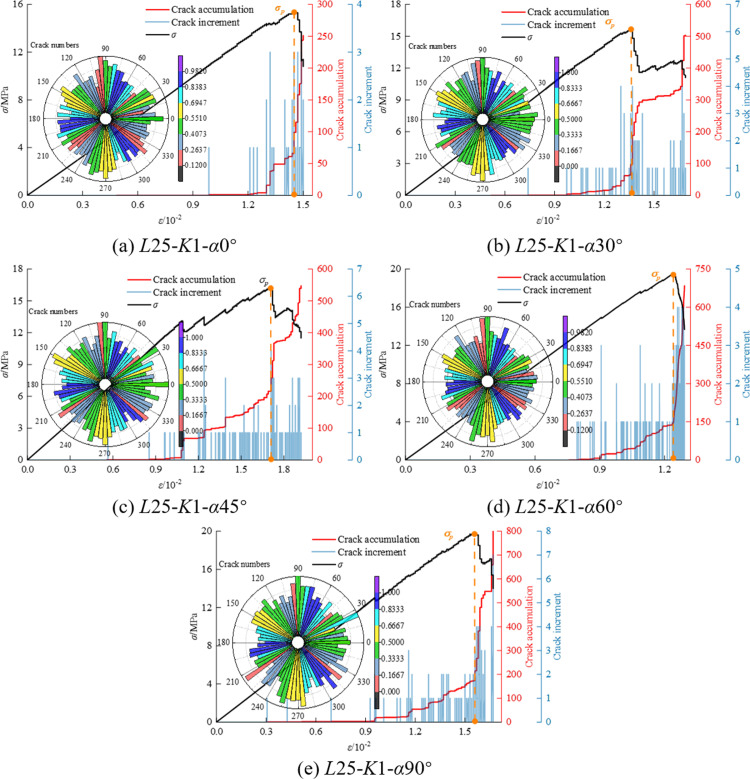
Crack number evolution curve and crack rose diagram of *K* = 1 mm.

**Fig 10:**
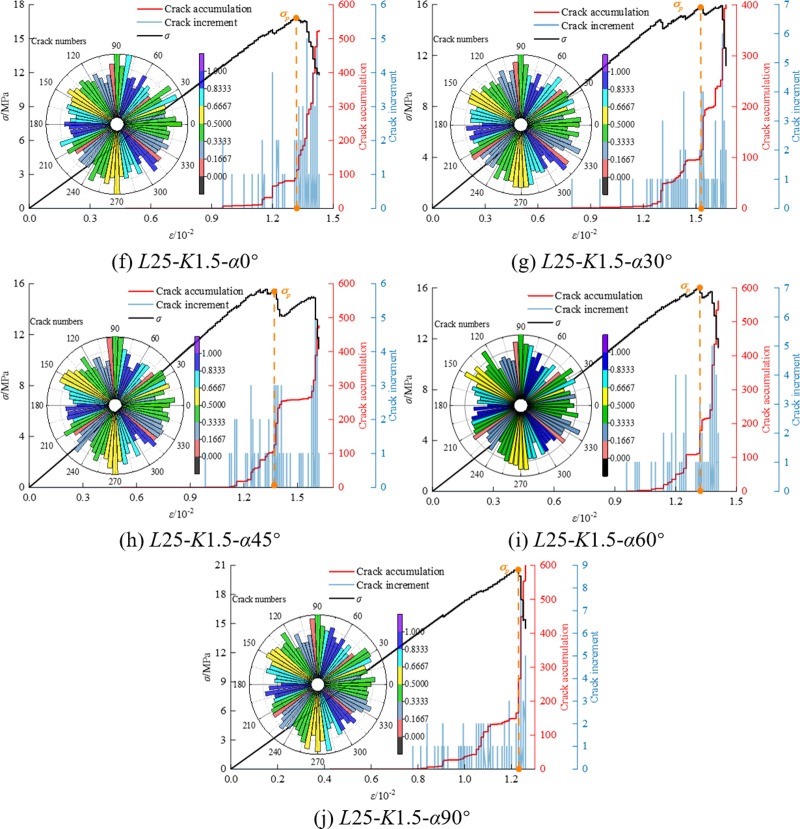
Crack number evolution curve and crack rose diagram of *K* = 1.5 mm.

**Fig 11:**
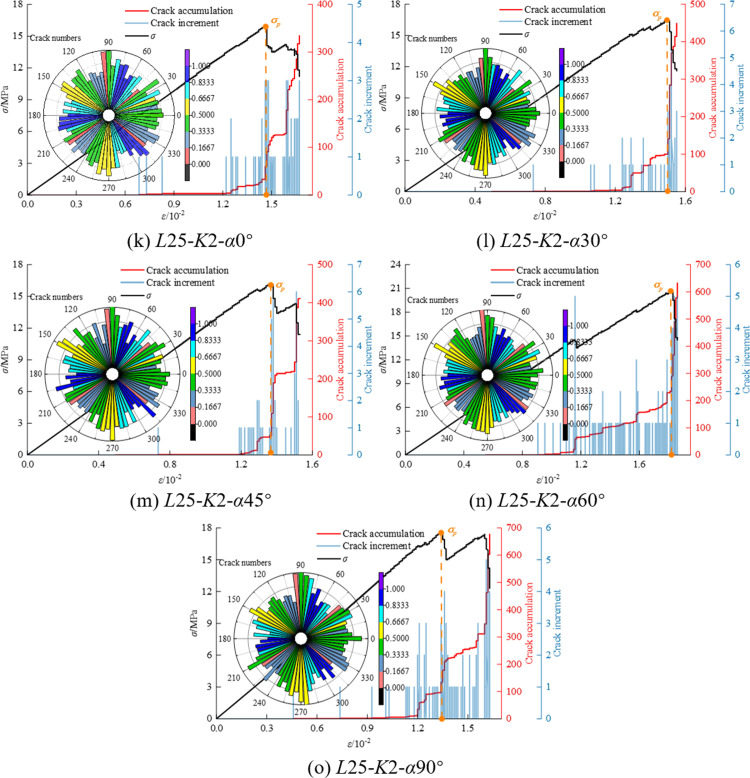
Crack number evolution curve and crack rose diagram of *K* = 2 mm.

In the process of axial loading, the numerical model is subjected to force in both directions, and the force is transmitted through particles. As the load gradually increases, when the ultimate strength of contact bonding is reached, the contact bonds between some particles break, generating cracks. As the load continues to increase, the fractures also expand synchronously, and the cumulative number of cracks reaches a new peak. The deterioration of the internal microstructure of the particles leads to further damage in the coal-rock composites, resulting in significant macroscopic damage. Fig 9, Fig 10 and Fig 11 show the crack number evolution curve and crack orientation accumulation diagram of the specimen under axial compression.

Through the crack azimuth accumulation diagram in Fig 9, Fig 10 and Fig 11, it is observed that the cracks generated in the sample are primarily concentrated at azimuth angles of 90° and 270°, with a high consistency in the distribution density of cracks at these angles. This indicates that the cracks predominantly propagate along the axial compression direction.

In the study of the crack growth process of coal and rock mass, the development of cracks can be compared with the four stages of the uniaxial experimental stress-strain curve, providing a detailed understanding of the changes in the number of cracks and their influence: ① In the initial compaction stage, the crack increment is 0. This is because the natural primary cracks in the coal rock mass are compacted under the action of external pressure, and no new cracks are generated. ② With the increase in loading, the coal-rock mass transitions from elastic deformation to the linear elastic deformation stage. At this point, new secondary cracks begin to form, based on the existing primary cracks. The total number of cracks reaches about 100, indicating the initiation of crack activity. ③ During the yield stage, stress within the coal-rock mass reaches the yield strength of the material, leading to accelerated crack growth. The primary fractures and the newly formed secondary fractures further propagate under stress, beginning to converge in specific regions, forming one or more main fracture zones. The number of cracks increases sharply, rising from hundreds to between 400 and 1000, and the crack distribution begins to exhibit localized characteristics. ④ In the failure stage, the number of cracks reaches the peak, and further crack expansion leads to the complete destruction of the coal-rock composites. Localized cracks are formed, and the structural integrity of coal and rock mass is lost.

### 4.2. Establishment of damage evolution constitutive equation

In order to further study the influence of fracture width and fracture dip angle on coal-rock composites, a damage constitutive model is constructed to accurately characterize the relationship between these parameters. During the loading process of the composite, the development of damage is closely related to the generation and propagation of cracks, which can be quantified by introducing the damage factor *D*. As a key index, the number of cracks effectively reflects the distribution of micro-cracks within the sample and the corresponding damage level [46]. Based on this, assuming that the specimen is in good condition before compression, the damage factor *D* can be defined as [47,48]:


$$D=\frac{N}{N_{0}}$$ (1)

where *D* represents the damage factor under axial compression, ranging from 0 to 1, where 0 indicates no damage and 1 indicates complete failure; *N* is the number of cracks generated due to specimen damage, and *N*_0_ is the total number of cracks that can potentially be generated in the specimen.

It is assumed that the relationship between the probability of failure of micro-units and the cumulative number of cracks under axial compression follows the Weibull distribution statistical probability model [49,50]. As the crack area size increase, the number of micro-units in the composite specimen under axial compression is:


$$f\left(N\right)=\left[\frac{0.5p_{3}N^{0.5}(p_{1}+p_{2}N^{0.5})-0.5p_{2}N^{0.5}(1+p_{3}N^{0.5})}{{\left(1+p_{3}N^{0.5}\right)}^{2}}\right]\text{exp}\left(\frac{p_{1}+p_{2}N^{0.5}}{1+p_{3}N^{0.5}}\right)$$ (2)

where *N* is the number of cracks in the specimen; *p*_1_ is the shape parameter, a dimensionless constant; *p*_2_ and *p*_3_ are the scale parameters in *N*^-0.5^, respectively.

It is assumed that there is the following relationship between the damage factor *D* and the number of cracks *N* generated at any time:


$$f\left(N\right)=\frac{dD}{dN}$$ (3)

According to Eq. (4), the damage factor *D* can be expressed as:


$$D=\int_{0}^{N}f\left(N\right)dN=1-\text{exp}\left(\frac{p_{1}+p_{2}N^{0.5}}{1+p_{3}N^{0.5}}\right)$$ (4)

If the cumulative number of cracks in the whole compression process of the specimen is *N*_*t*_, *N*(*t*) represents the cumulative number of cracks at any time in the axial compression process of the specimen, then the relationship between *N*_*t*_ and *N*(*t*) is:


$$N(t)=N_{t}\int_{0}^{N}f\left(N\right)dN=1-\text{exp}\left(\frac{p_{1}+p_{2}N^{0.5}}{1+p_{3}N^{0.5}}\right)$$ (5)

Therefore, the composites of Eq. (4) and Eq. (5) is transformed, and the damage factor expression of coal-rock composites specimen can be obtained:


$$\frac{N(t)}{N_{t}}=D=1-\text{exp}\left(\frac{p_{1}+p_{2}N^{0.5}}{1+p_{3}N^{0.5}}\right)$$ (6)

Considering that the damage factor *D*, calculated based on the number of cracks, is highly consistent with the prediction results of the Weibull model, it is reasonable to use the number of cracks to characterize the damage evolution. Based on the effective stress theory presented in references [51, 52], the stress-strain relationship of coal rock damage can be expressed as follows:


$$\sigma =E\varepsilon \cdot \left(1-D\right)$$ (7)

where *ε* is the strain of the samples and *E* is the elastic modulus of the samples.

By substituting Eq. (6) into Eq. (7), the damage evolution constitutive equation can be calculated as follows:


$$\sigma =E\varepsilon \cdot \text{exp}\left(\frac{p_{1}+p_{2}N^{0.5}}{1+p_{3}N^{0.5}}\right)$$ (8)

The stress-strain, elastic modulus and fracture number are substituted into Eq. (8), and the parameters *p*_1_, *p*_2_ and *p*_3_ are solved by nonlinear fitting.

**Fig 12:**
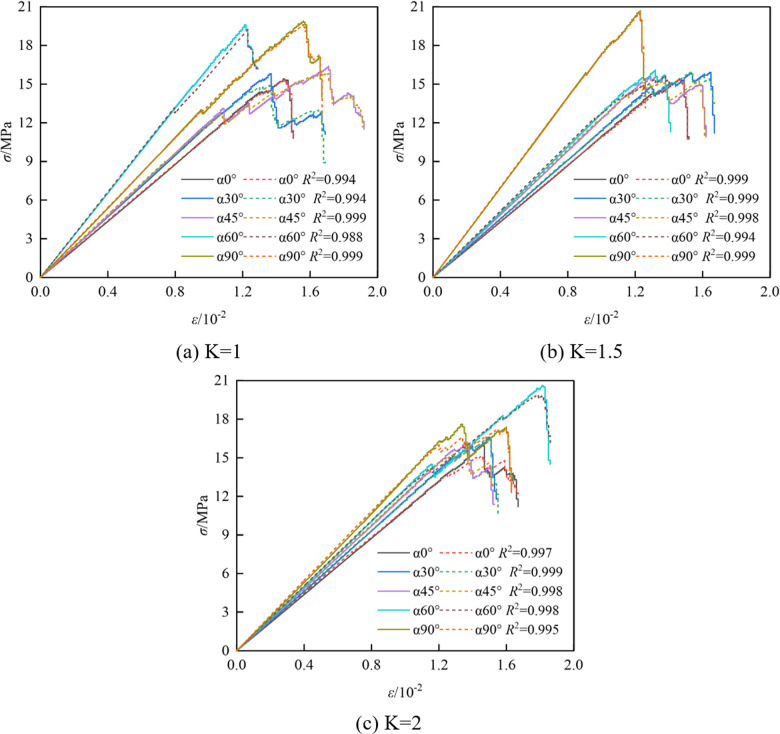
Verification of the constitutive model.

The specific parameters in the damage evolution constitutive equation of the coal-rock composites are listed, and the verification results of the constitutive model are shown in Fig 12. The solid line in the figure represents the simulated value, while the dashed line represents the theoretical value. It can be observed that, although the simulated curve exhibits fluctuates near the peak, the stress-strain curve calculated by the constitutive equation is generally consistent with the simulated curve in terms of trend, and maintains a good fit in the post-peak stage.

According to the equations, the fracture area *S* and the fracture dip angle *α* can be used as independent variables, while the parameters *p*_1_, *p*_2_ and *p*_3_ are dependent variables that can be used to fit the coal-rock composites. The relationship between these variables is expressed in the following form:


$$\{\begin{array}{c} \begin{array}{c} \sigma =E\varepsilon \cdot \exp\left(\frac{p_{1}+p_{2}N^{0.5}}{1+p_{3}N^{0.5}}\right)\\ p_{1}=\frac{0.0003S-1.23S^{2}+9.47S^{3}-0.027\alpha +2.31}{1+0.00011S-0.012\alpha } \end{array}\\ \begin{array}{c} p_{2}=\frac{9.3S^{2}\times 10^{7}-6.19S\times 10^{10}-9.28\alpha \times 10^{12}+9.25\times 10^{12}}{1+1.27S\times 10^{10}+8.47S^{2}\times 10^{7}+1.1\alpha \times 10^{14}}\\ p_{3}=\frac{0.133S-7.75S^{2}-0.00012\alpha -0.023}{1-0.00056S-0.044\alpha +0.00065\alpha^{2}} \end{array} \end{array}$$ (9)

### 4.3 Damage evolution analysis of cracked coal-rock composites

**Fig 13:**
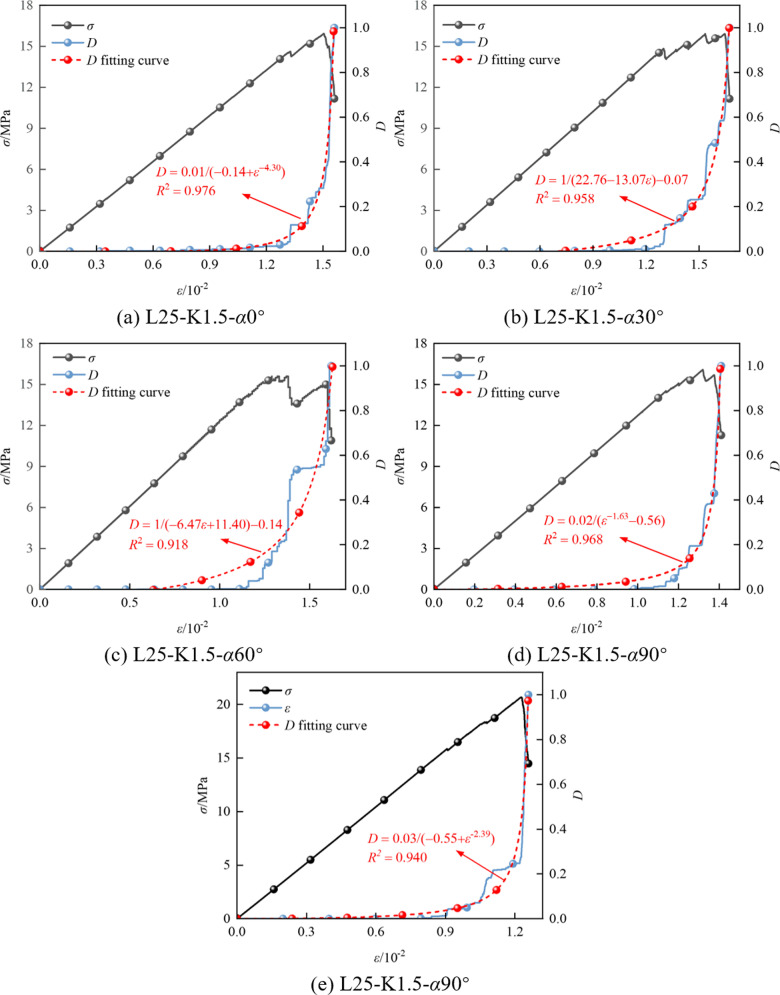
Damage evolution of specimens with *K* = 1.5 mm.

According to the constitutive relation in Eq. (9), the damage evolution equation of the cracked coal-rock composites, considering sensitivity factors such as the fracture inclination angle and fracture area, can be effectively characterized, as shown in Eq. (10). Thus, the damage evolution curve of the cracked coal-rock composites is obtained. Taking the *K* =  1.5 mm model as an example, the evolution curve is shown in Fig 13.

It can be observed that: ① The damage factor *D* increases nonlinearly with the strain, and the fitting curve's *R*^2^ value is close to 1, indicating a good fitting effect. ② Under different inclination angles, the damage curve of coal-rock composites is consistent. The increase in axial pressure leads to the formation of new cracks, aggravating the damage, which then attenuates after the peak value. ③ In the initial loading stage, the slope of the damage curve is 0, indicating no cracks. As the loading progresses, the damage factor *D* increases slowly, and the cracks expand rapidly. In the peak stress stage, the damage becomes significant, accounting for 60%-80% of the failure process. The cracks connect, causing a sharp drop in stress. ④ Taking the specimen with a 45° inclination as a demarcation, it can be observed that the strain at the peak stress damage stage for the specimens with 0° and 30° inclination angles is higher than that for specimens with 60° and 90° inclination angles. This indicates that the specimens with larger inclination angles experience more severe damage and faster degradation. This also shows that increasing the fracture inclination angle can enhance the compressive performance of the coal-rock composites, but it accelerates the instability process of the coal-rock composite structure.


$$\{\begin{array}{c} \begin{array}{c} D=1-\exp\left(\frac{p_{1}+p_{2}N^{0.5}}{1+p_{3}N^{0.5}}\right)\\ p_{1}=\frac{0.0003S-1.23S^{2}+9.47S^{3}-0.027\alpha +2.31}{1+0.00011S-0.012\alpha } \end{array}\\ \begin{array}{c} p_{2}=\frac{9.3S^{2}\times 10^{7}-6.19S\times 10^{10}-9.28\alpha \times 10^{12}+9.25\times 10^{12}}{1+1.27S\times 10^{10}+8.47S^{2}\times 10^{7}+1.1\alpha \times 10^{14}}\\ p_{3}=\frac{0.133S-7.75S^{2}-0.00012\alpha -0.023}{1-0.00056S-0.044\alpha +0.00065\alpha^{2}} \end{array} \end{array}$$ (10)

## 5 Conclusions and discussion

This study systematically analyzed the mechanical properties and crack damage evolution of coal-rock composites containing cracks under uniaxial compression, through both laboratory uniaxial compression experiments and numerical simulations. A constitutive equation for crack damage evolution was developed. The results demonstrate that the presence of fractures significantly influences the mechanical behavior of coal-rock composites, with particular emphasis on the role of crack inclination in regulating the compressive strength and failure mode of the composite material.

1. In uniaxial compression experiments on different specimens, sandstone exhibited the highest peak strength and elastic modulus, followed by pure coal, while the macro-mechanical parameters of the coal-rock composites were intermediate. The strength and stiffness of coal-rock composites containing cracks were significantly lower than those without cracks due to the presence of cracks. The crack angle and width exerted a significant influence on the mechanical behavior of the coal-rock composites: the strength and elastic modulus of *K*1 and *K*1.5 gradually increased with the increase of the inclination angle, while *K*2 exhibited a nonlinear change of increasing and then decreasing.
2. The failure modes of the coal-rock composites are primarily dominated by the coal component, exhibiting bulk failure, which indicates that the failure process is governed by the combined action of tension and shear. This emphasizes the need to consider the interaction of these two damage mechanisms in the assessment of structural stability. Analysis of the crack evolution curves and radial accumulation diagrams shows that the cracks are mainly concentrated at azimuthal angles of 90° and 270°, indicating that they primarily propagate along the axial compression direction. With the increase in crack inclination angle, the crack propagation path changes significantly, resulting in a reduced damage degree in the rock component and an intensified damage degree in the coal component, further highlighting the crucial role of the crack inclination angle in regulating the damage process of coal-rock composites.
3. The ontological equations and damage evolution curves based on the number of cracks show that there is a nonlinear relationship between the damage factor and the strain, and the shape of the damage curves remains unaffected by changes in the crack inclination angle. Taking the 45° inclination specimen as the demarcation, it can be observed that the strain at the peak stress stage for the specimens with 0° and 30° inclination angles is lower than that of the specimens with 60° and 90° inclination angles, indicating that while an increase in the crack inclination angle can improve the compressive performance of the coal-rock composites, it accelerates the process of structural instability.

These findings provide valuable insights for mine crack management. In practical applications, mining engineers should pay particular attention to the distribution and inclination of cracks, especially in regions with larger crack inclinations. More precise crack control and reinforcement measures should be implemented in these areas. For instance, enhanced crack monitoring and analysis, as well as techniques like grouting, could be employed to mitigate crack propagation. Moreover, adaptive support designs should be adopted in high-risk areas to improve the overall stability of the mine structure.

We recommend that when designing mine support structures, the characteristics of fractures particularly crack inclination, distribution, and failure modes be comprehensively considered. Suitable support materials, tailored to the fracture characteristics of coal-rock composites, should be selected, with adjustments made according to variations in crack inclination to ensure that the support system can effectively address different failure modes. Additionally, the monitoring and maintenance of the support structure should be strengthened to mitigate the risks associated with crack propagation and structural instability.

## Supporting information

S1 Data Raw data for figures. (ZIP)

S1 Fig All figures presented in the study. (ZIP)
