# Supplementary figures and images for: Study on the damage constitutive characteristics of coal-rock composites under uniaxial compression: Influence of prefabricated crack angle and geometric dimensions
Source: PLoS One. 2025 Mar 5;20(3):e0316586. doi: 10.1371/journal.pone.0316586 (PMC11882100; doi:10.1371/journal.pone.0316586)

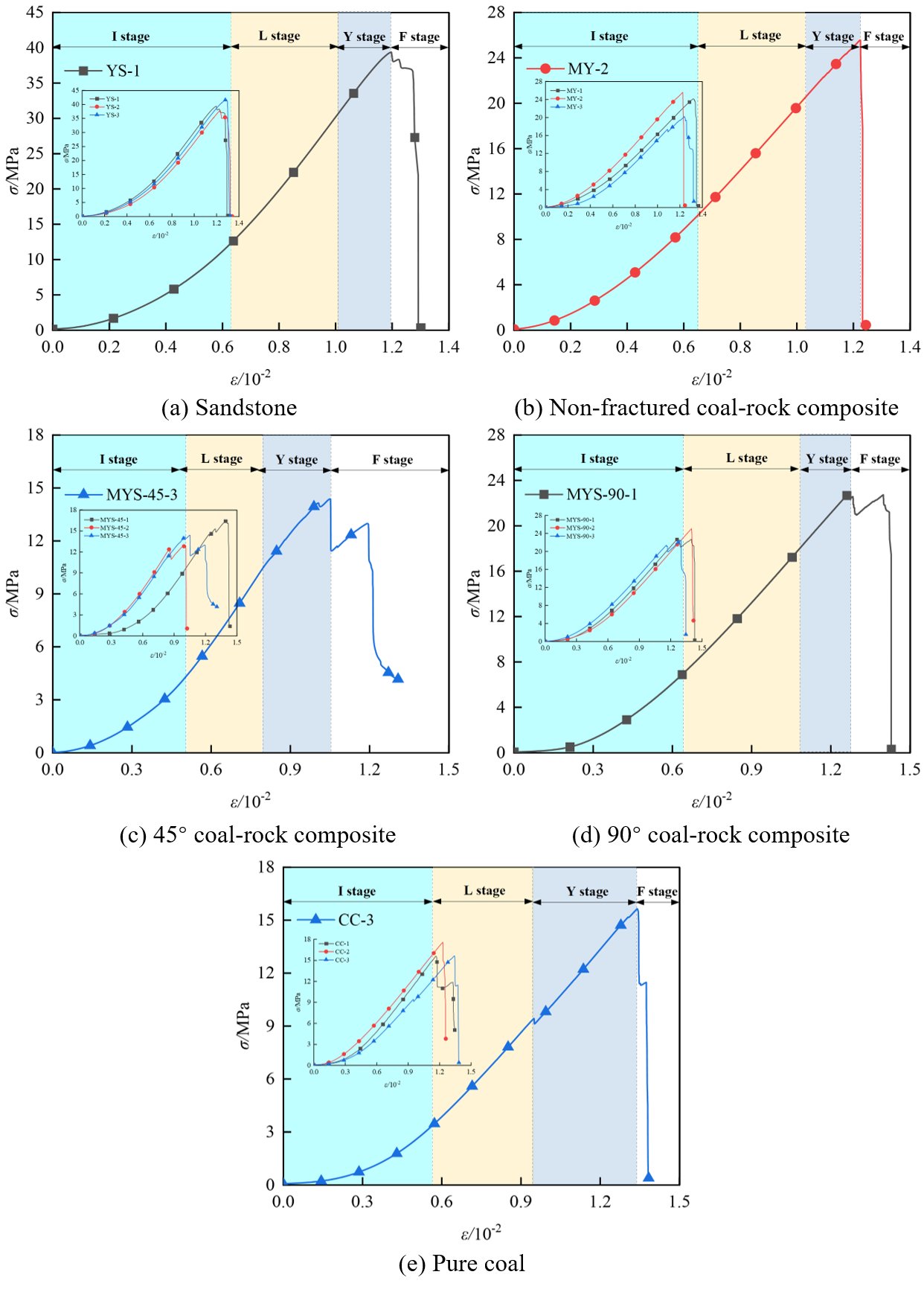

Supplement: S1 Fig — (ZIP) [file pone.0316586.s002.zip › PACE Corrected/Fig 1.tif]

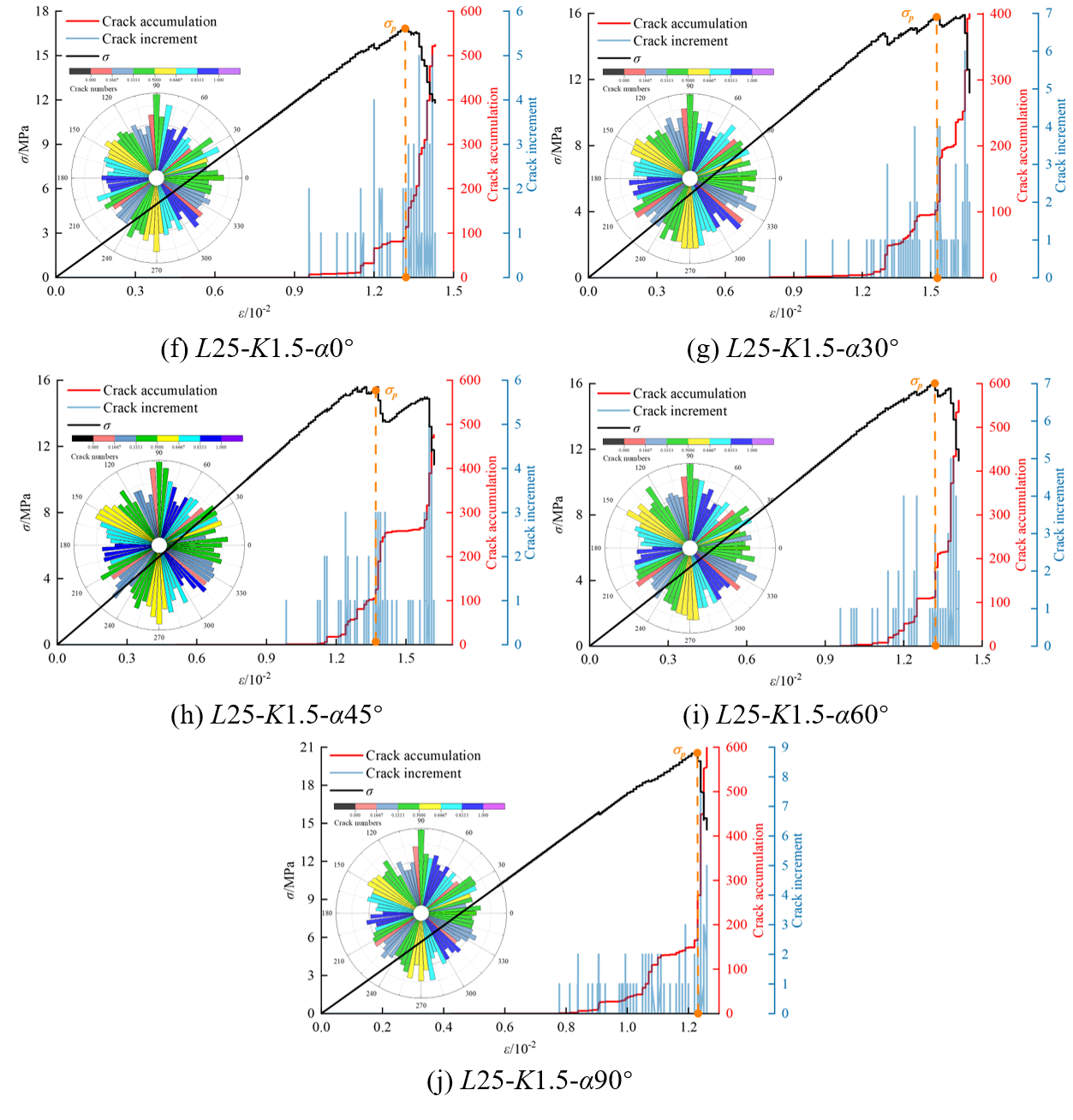

Supplement: S1 Fig — (ZIP) [file pone.0316586.s002.zip › PACE Corrected/Fig 10.tif]

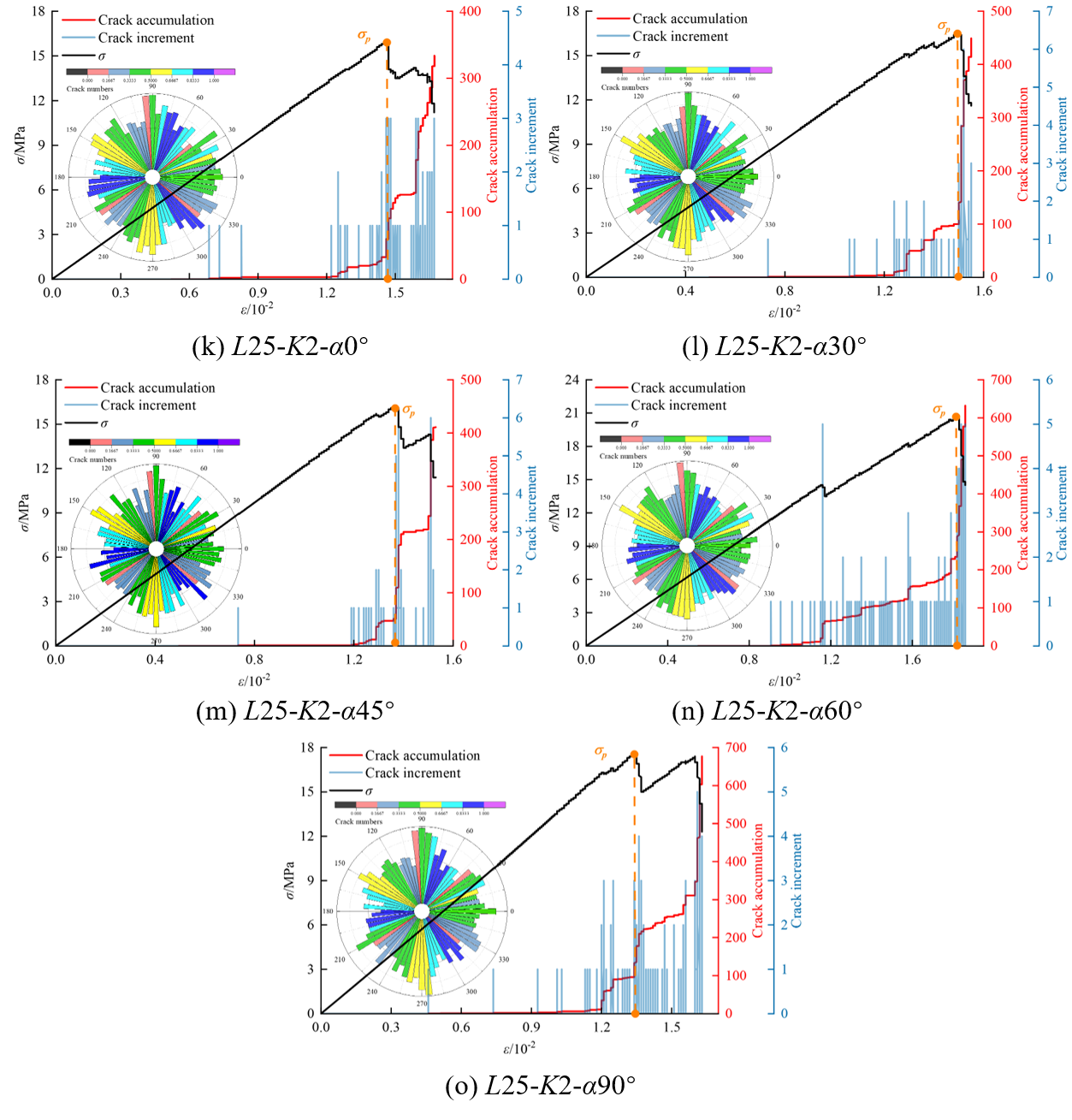

Supplement: S1 Fig — (ZIP) [file pone.0316586.s002.zip › PACE Corrected/Fig 11.tif]

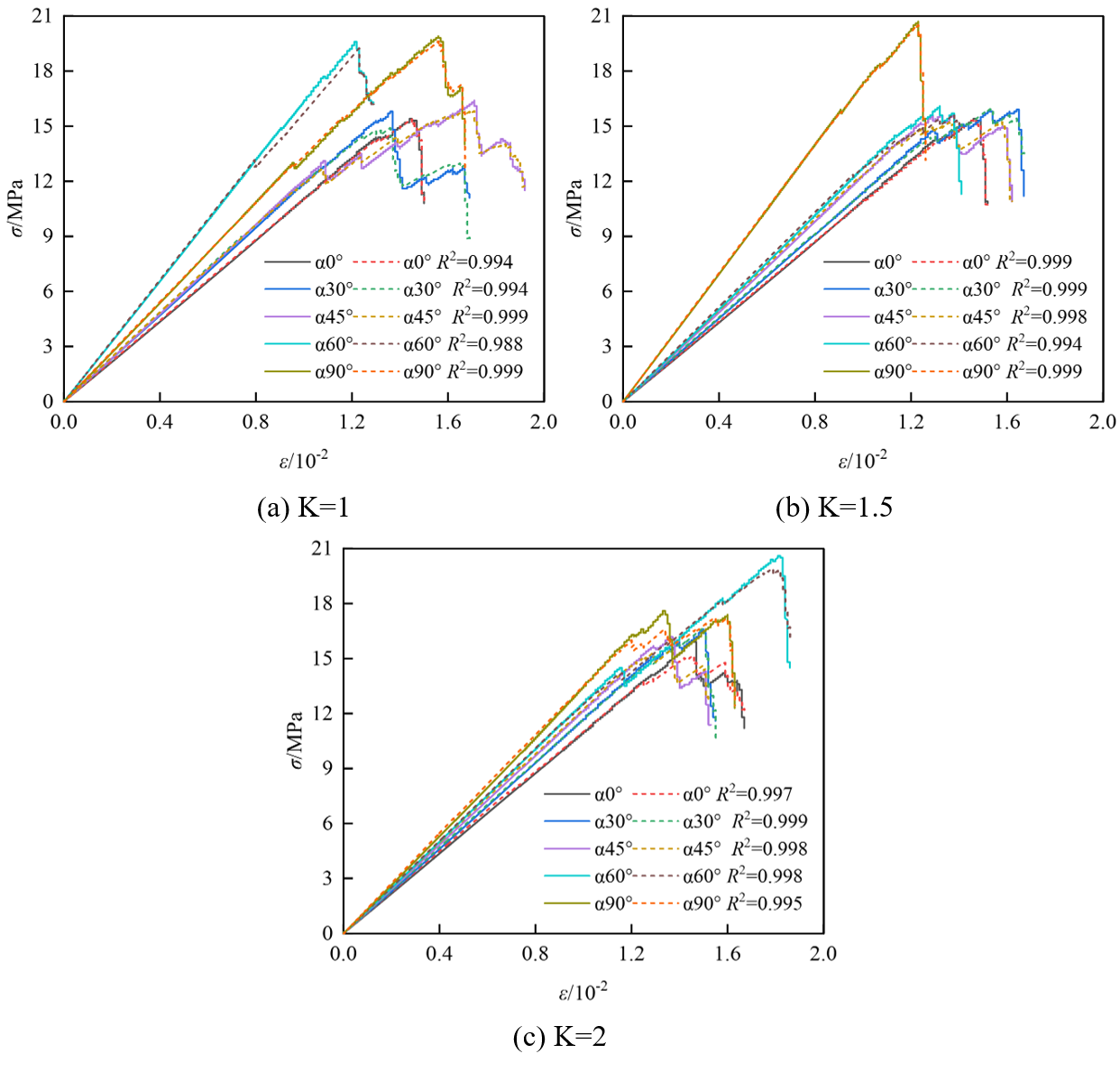

Supplement: S1 Fig — (ZIP) [file pone.0316586.s002.zip › PACE Corrected/Fig 12.tif]

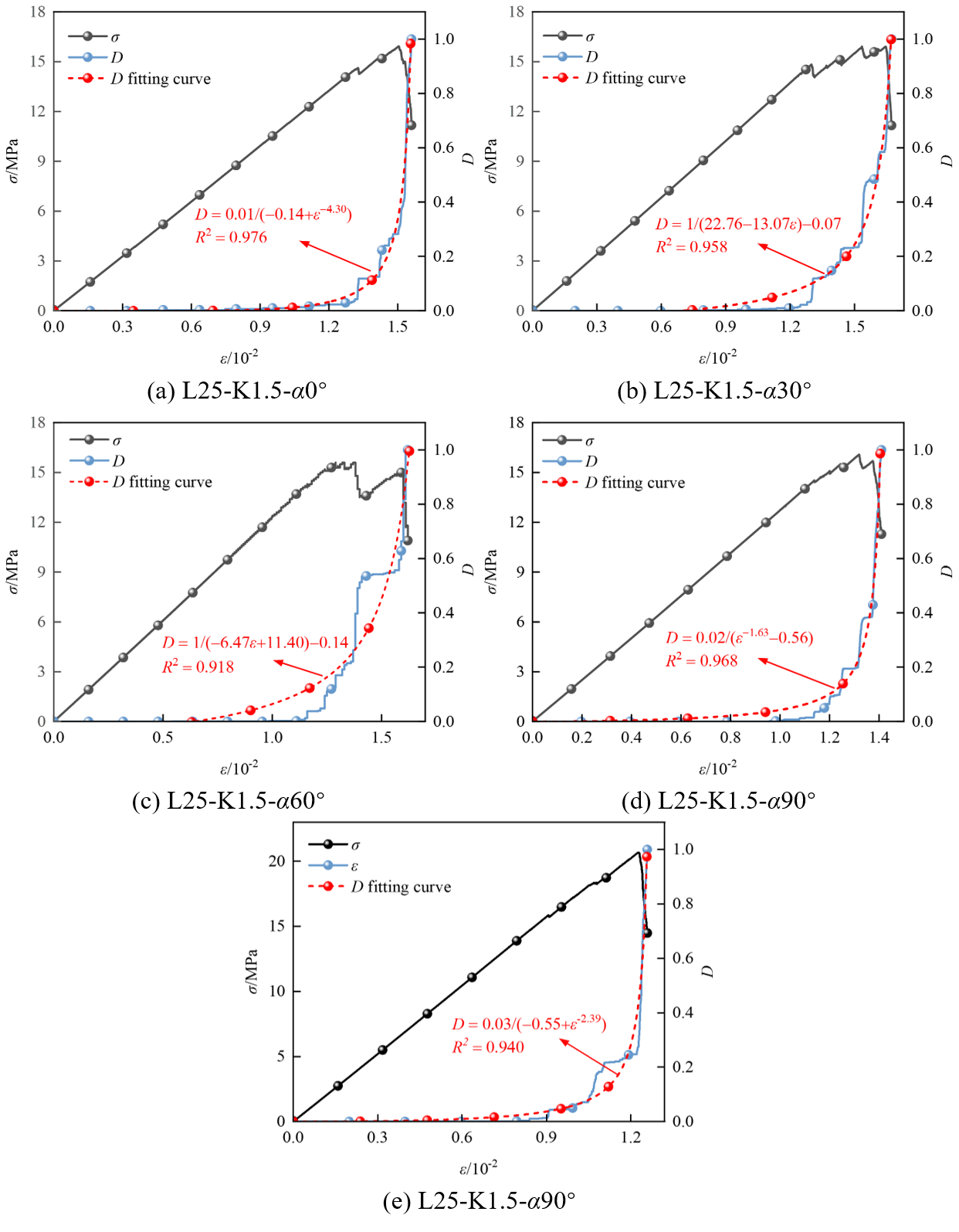

Supplement: S1 Fig — (ZIP) [file pone.0316586.s002.zip › PACE Corrected/Fig 13.tif]

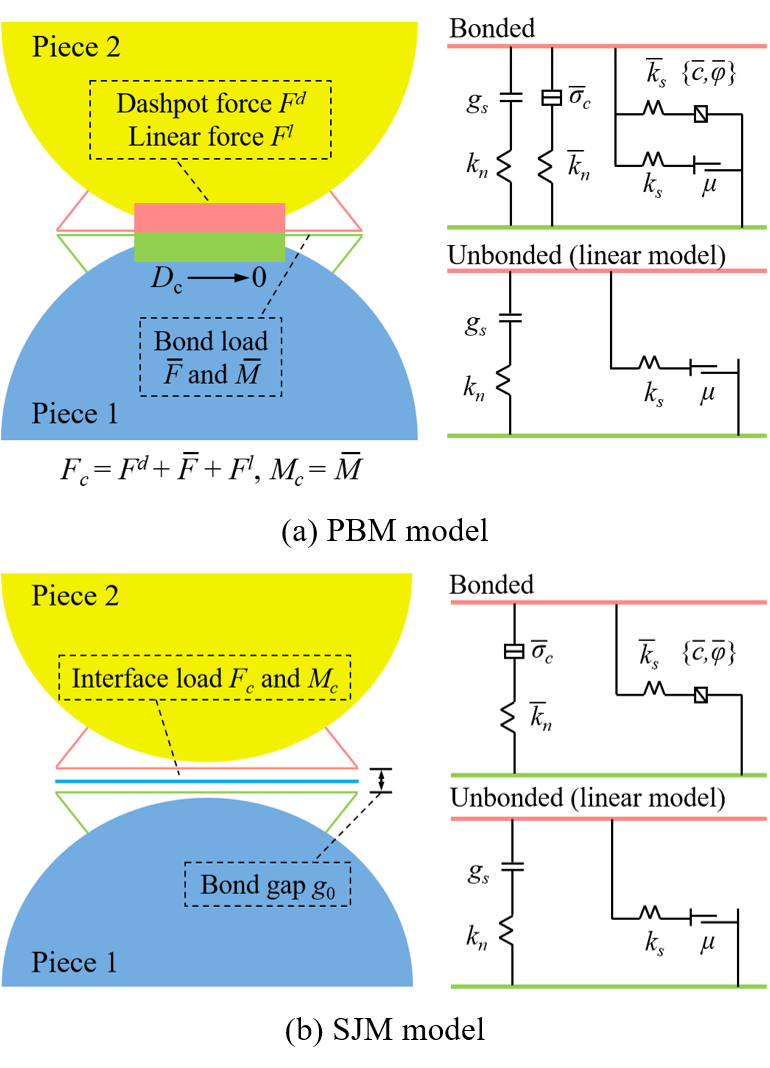

Supplement: S1 Fig — (ZIP) [file pone.0316586.s002.zip › PACE Corrected/Fig 2.tif]

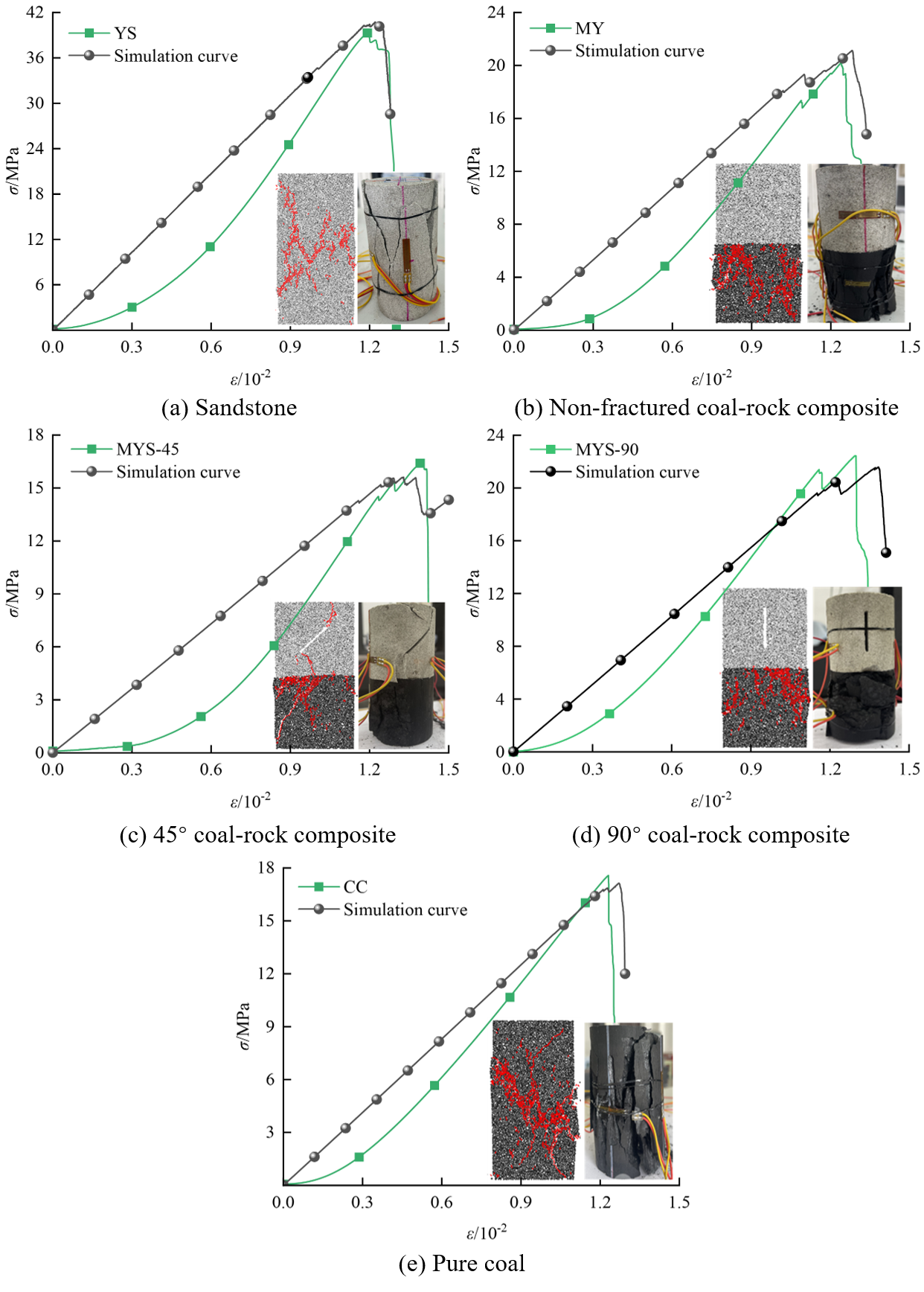

Supplement: S1 Fig — (ZIP) [file pone.0316586.s002.zip › PACE Corrected/Fig 3.tif]

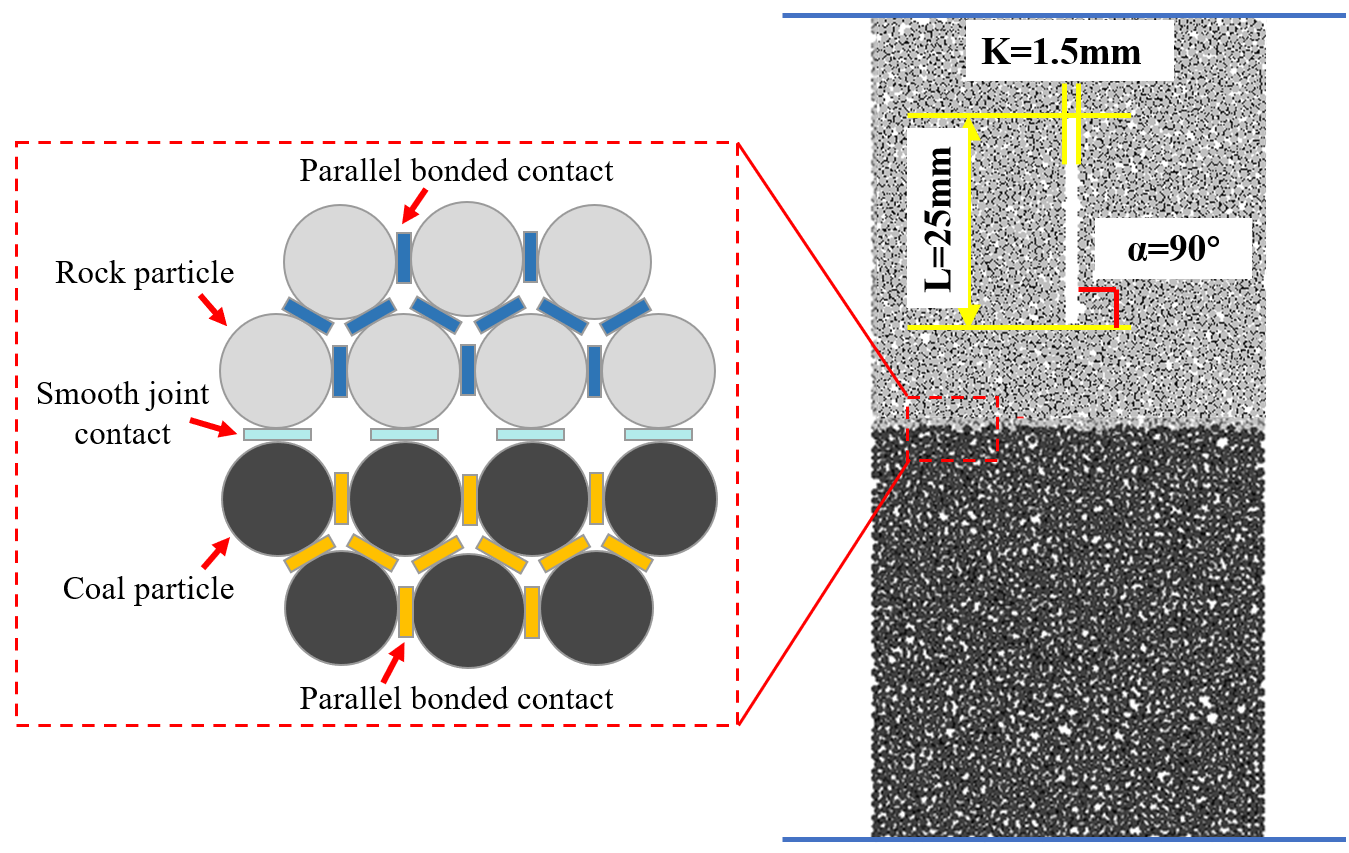

Supplement: S1 Fig — (ZIP) [file pone.0316586.s002.zip › PACE Corrected/Fig 4.tif]

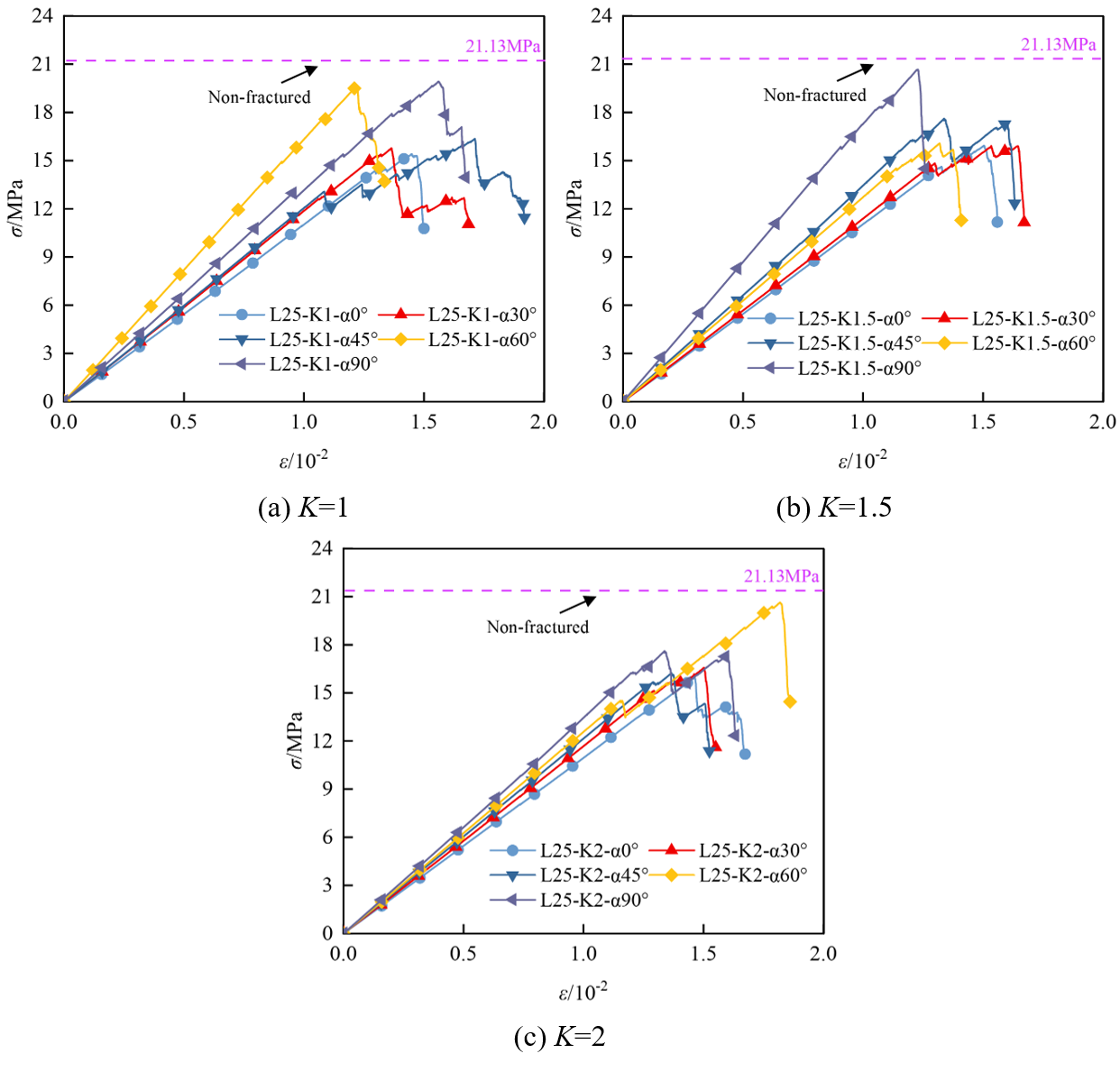

Supplement: S1 Fig — (ZIP) [file pone.0316586.s002.zip › PACE Corrected/Fig 5.tif]

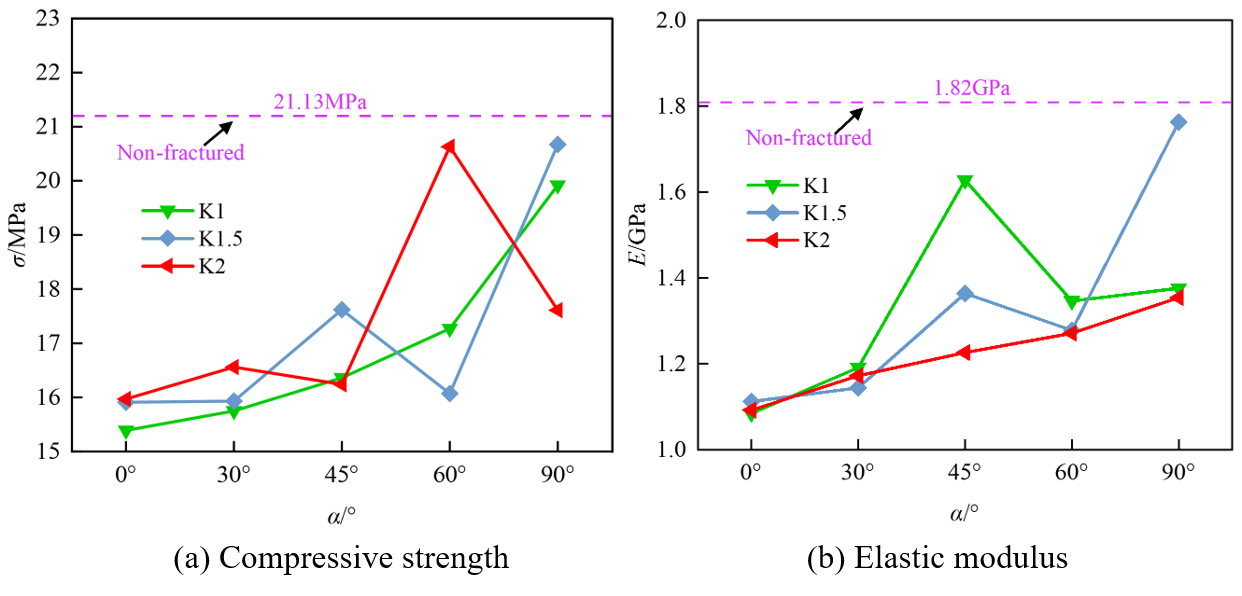

Supplement: S1 Fig — (ZIP) [file pone.0316586.s002.zip › PACE Corrected/Fig 6.tif]

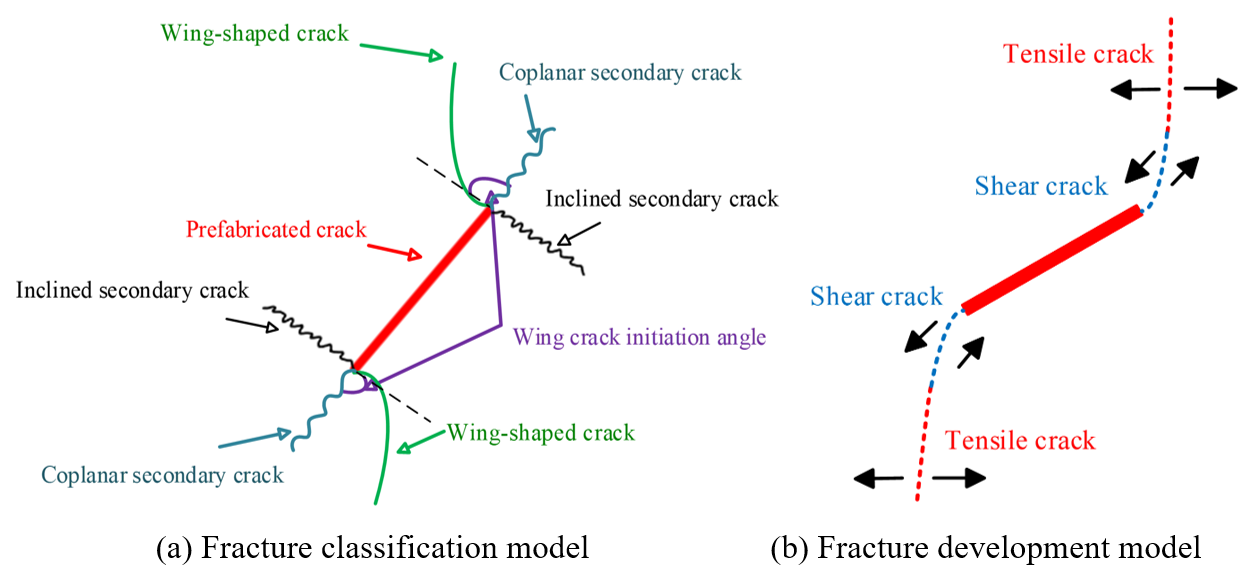

Supplement: S1 Fig — (ZIP) [file pone.0316586.s002.zip › PACE Corrected/Fig 7.tif]

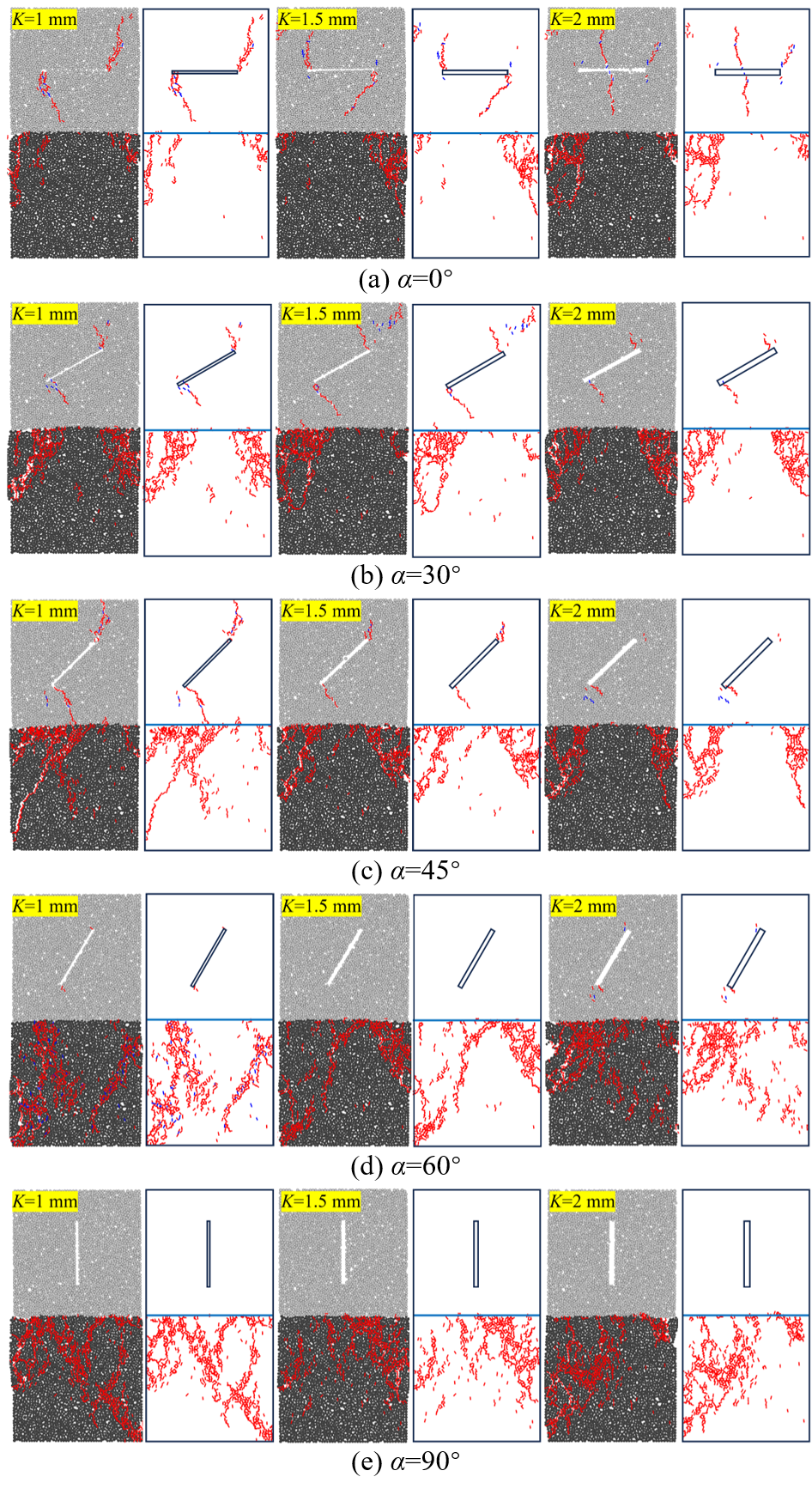

Supplement: S1 Fig — (ZIP) [file pone.0316586.s002.zip › PACE Corrected/Fig 8.tif]

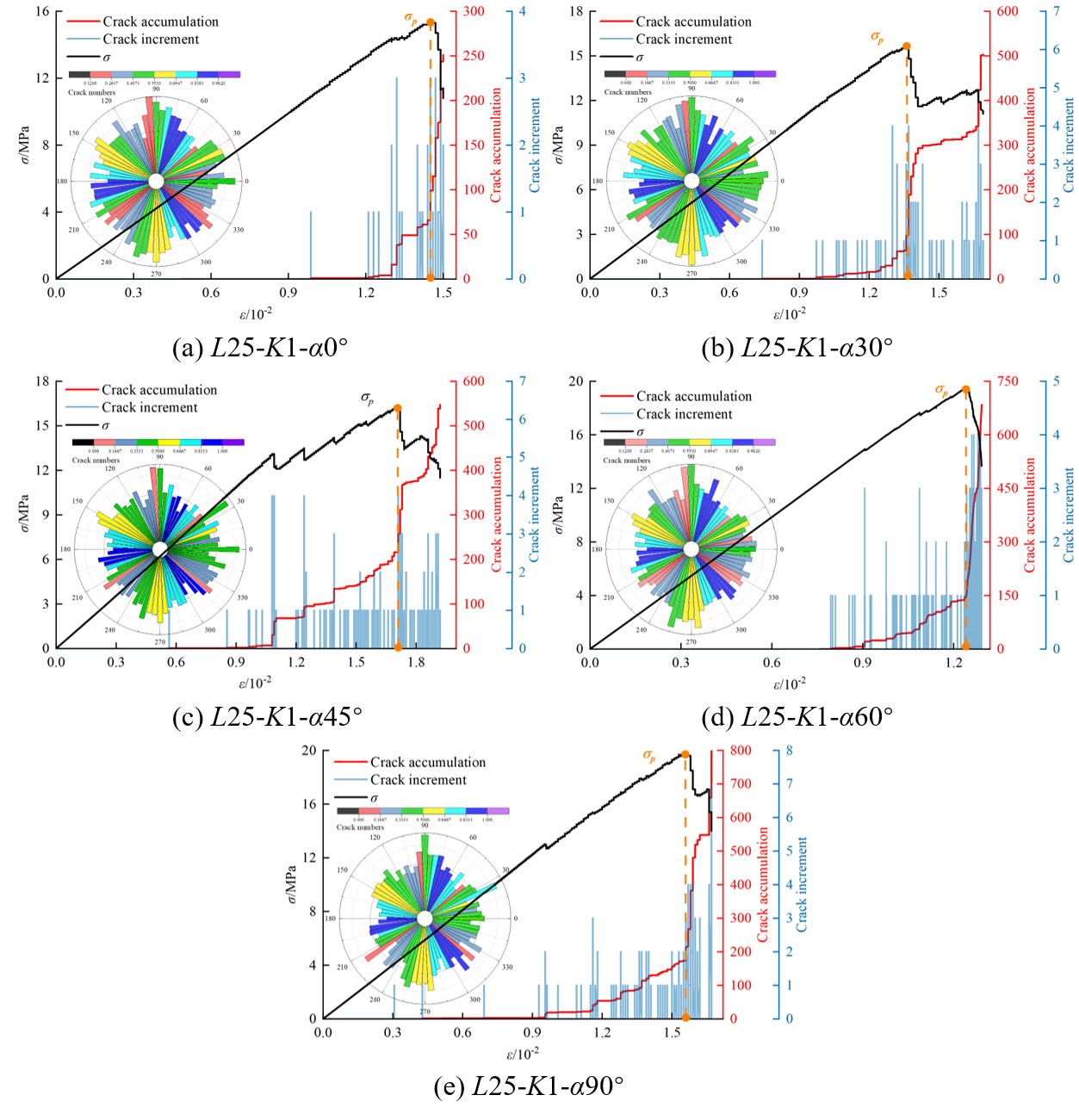

Supplement: S1 Fig — (ZIP) [file pone.0316586.s002.zip › PACE Corrected/Fig 9.tif]
